# Identifying Targets for Antibiotic Use for the Management of Carbapenem-Resistant *Acinetobacter baumannii* (CRAb) in Hospitals—A Multi-Centre Nonlinear Time-Series Study

**DOI:** 10.3390/antibiotics11060775

**Published:** 2022-06-07

**Authors:** Zainab Said Al-Hashimy, Barbara R. Conway, Mubarak Al-Yaqoobi, Faryal Khamis, Ghalib Zahran Al Mawali, Aisha Mahad Al Maashani, Yaqoob Said Al Hadhrami, Said Salim Al Alawi, Mohammed Said Al Mamari, William J. Lattyak, Elizabeth A. Lattyak, Motasem Aldiab, Ian Gould, José-María López-Lozano, Mamoon A. Aldeyab

**Affiliations:** 1 Directorate of Pharmacy and Medical Stores, Khawlah Hospital, Muscat P.O. Box 90, Oman; zainab.alhashimy@outlook.com; 2 Department of Pharmacy, School of Applied Sciences, University of Huddersfield, Huddersfield HD1 3DH, UK; b.r.conway@hud.ac.uk; 3 Institute of Skin Integrity and Infection Prevention, University of Huddersfield, Huddersfield HD1 3DH, UK; 4 Directorate of Laboratories, Department of Microbiology, Khawlah Hospital, Muscat P.O. Box 90, Oman; mubarak18@gmail.com; 5 Adult Infectious Disease, Department of Medicine, Royal Hospital, Muscat P.O. Box 1331, Oman; khami001@gmail.com; 6 Pharmaceutical Care Department, Royal Hospital, Muscat P.O. Box 1331, Oman; gmaawaly@gmail.com; 7 Department of Pharmacy and Medical Store, As Sultan Qaboos Hospital, Salalah P.O. Box 98, Oman; amsa4383@gmail.com; 8 Department of Pharmacy and Medical Store, Nizwa Hospital, Nizwa P.O. Box 1222, Oman; yaqoob_999@hotmail.com; 9 Department of Pharmacy and Medical Store, Sur Hospital, Sur P.O. Box 966, Oman; cheedy7801@gmail.com; 10 Department of Pharmacy and Medical Store, Ibra Hospital, Ibra P.O. Box 162, Oman; almammri333@hotmail.com; 11 Scientific Computing Associates Corp., River Forest, IL 60305, USA; blattyak@scausa.com (W.J.L.); elizabeth.lattyak@sbcglobal.net (E.A.L.); 12 Department of Computing, British Columbia Institute of Technology, Vancouver, BC V6B 3H6, Canada; motasem.aldiab@gmail.com; 13 Medical Microbiology Department, Aberdeen Royal Infirmary, Aberdeen AB25 2ZN, UK; i.m.gould@abdn.ac.uk; 14 Biomedical Research Institute of Murcia (IMIB-Arrixaca), 30120 Murcia, Spain; josemaria.lopezl@um.es; 15 Research Group on Health Sciences Data Analysis, Universidad de Murcia, 30003 Murcia, Spain

**Keywords:** antibacterial agents, carbapenem-resistant Enterobacteriaceae, *Acinetobacter baumannii*, antibiotic stewardship, hand sanitizers, alcohol-based hand rub, non-linear time-series analysis, thresholds, antibiotic consumption

## Abstract

Solutions are needed to inform antimicrobial stewardship (AMS) regarding balancing the access to effective antimicrobials with the need to control antimicrobial resistance. Theoretical and mathematical models suggest a non-linear relationship between antibiotic use and resistance, indicating the existence of thresholds of antibiotic use beyond which resistance would be triggered. It is anticipated that thresholds may vary across populations depending on host, environment, and organism factors. Further research is needed to evaluate thresholds in antibiotic use for a specific pathogen across different settings. The objective of this study is to identify thresholds of population antibiotic use associated with the incidence of carbapenem-resistant *Acinetobacter baumannii* (CRAb) across six hospital sites in Oman. The study was an ecological, multi-centre evaluation that involved collecting historical antibiotic use and CRAb incidence over the period from January 2015 to December 2019. By using non-linear time-series analysis, we identified different thresholds in the use of third-generation cephalosporins, piperacillin-tazobactam, aminoglycoside, and fluoroquinolones across participating hospitals. The identification of different thresholds emphasises the need for tailored analysis based on modelling data from each hospital. The determined thresholds can be used to set targets for each hospital AMS, providing a balance between access to these antibiotics versus controlling CRAb incidence.

## 1. Introduction

Antibiotics are one of the essential medical interventions that reduce human morbidity and mortality; however, the overuse and misuse of antibiotics contribute to the spread of resistance and threaten many achievements of modern medicine [1,2,3,4]. A recent comprehensive analysis showed that the global burden associated with drug-resistant infections in 2019 was an estimated 4.95 million deaths, of which 1.27 million deaths were directly attributable to drug resistance [3]. The link between antibiotic use and the subsequent selection and spread of antibiotic resistance is well established [1,5,6,7,8]. This link contributes to reducing their therapeutic effect. Considering a lack in newly approved antibiotics, it is vital to conserve the effectiveness of existing antibiotics. Solutions are needed to inform (AMS) to balance access to effective antimicrobials with the need to control antimicrobial resistance [9,10,11,12].

The antimicrobial resistance (AMR) situation in Oman is similar to the observed AMR globally [13]. In 2017, the Ministry of Health (MOH) in Oman launched the Oman antimicrobial surveillance system (OMASS) as part of a national action plan for the containment of antimicrobial resistance. The report showed the rate of bacterial resistance to antibiotics in hospitals in Oman. The surveillance included six hospitals in Oman [14].

OMASS reported that the CRAb were resistant to imipenem and meropenem in 73% and 81% of the cases, respectively. Also, *Acinetobacter baumannii* has revealed high resistance to aminoglycosides, the β-lactam group of antibiotics, and fluoroquinolones. In 2017, the carbapenem-resistant Enterobacteriaceae (CRE) rate in Oman was 12/1000 of patients with bacteraemia, and the mortality rate was 32% [14]. In the following year, the CRAb rate was increased to 75.5% for imipenem and 84% for meropenem [15].

The rate of carbapenem resistance to gram-negative bacteria tended to rise during the last 20 years. In one study, which was done in a teaching hospital in Oman, it was found that the CRAB rate increased from 67% to 86% between 2007–2016. The study also showed that 30-day all-cause mortality was high in CRAb (40.2%) [16].

WHO developed hand hygiene care guidelines which helped to implement good practice and reduce the transmission of nosocomial infections to the patient and health care provider [17]. Many studies showed that alcohol-based hand rubs reduce the rate of transmission of the infection to the patients. Health-care-associated infections (HAIs) can be prevented by implementing hand hygiene strategies that involved alcohol-based hand rubs, which will reduce AMR and mortality among admitted patients [18,19].

Studies have previously reported on the development of non-linear relationships between antibiotic use and resistance to propose thresholds of antibiotic use beyond which resistance would be triggered [1,9,10,11,20,21,22]. A recently published study determined a threshold in third-generation cephalosporin and carbapenem use, and the incidence of CRAb in hospitals in Jordan [11]. It is anticipated that thresholds may vary across populations depending on host, environment, and organism factors [9,10], therefore it would be useful to evaluate thresholds for specific pathogens across different settings. However, this is the first study to measure thresholds between antibiotic use and CRAb incidence across different hospitals. CRAb infections are associated with high mortality and few treatment options; addressing AMR required coordinated national and international efforts [23,24,25].

The aim of this study was to identify thresholds of population antibiotic use associated with the incidence of CRAb in six hospitals. The six hospitals, located in Oman, were reasonably similar in terms of being country-specific but varied in sizes, specialties, volumes of antibiotics, and CRAb incidence rates. In addition, we aimed to measure thresholds in associations between the use of alcohol-based hand rub (ABHR) and CRAb incidence rates in participating hospitals.

## 2. Results

The average monthly CRAb incidence (normalised per 100 occupied bed days (OBD) for participating hospitals was as follows: Royal Hospital (0.030), Khawlah Hospital (0.108), As Sultan Qaboos Hospital (0.140), Nizwa Hospital (0.087), Sur Hospital (0.187), and Ibra Hospital (0.104). The average monthly OBD for each hospital is shown in Table S1. The use of certain antibiotics was positively associated with the incidence of CRAb cases. The use of third-generation cephalosporins was associated with CRAb cases in the Royal Hospital and Nizwa Hospital, while piperacillin-tazobactam was identified in Khawlah Hospital, Sur Hospital, and Ibra Hospital. The use of aminoglycoside was associated with CRAb cases in Khawlah Hospital, As Sultan Qaboos Hospital, and Nizwa Hospital, while fluoroquinolone use was identified in As Sultan Qaboos Hospital (Table 1). The median use of these antibiotics and the determined lag effects (delay necessary to observe the effect in months) in each hospital are shown in Table 1.

By using non-linear time-series analysis, we identified different thresholds for third-generation cephalosporins, piperacillin-tazobactam, aminoglycoside, and fluoroquinolones across the participating hospitals. For the Royal Hospital, the use of third-generation cephalosporins should not exceed 6 defined daily dose (DDD)/100 OBD. In addition, ABHR reduced CRAb incidence up to 5.1 L/100 OBD, with no further impact beyond this threshold. For Sur Hospital, we identified a threshold for piperacillin-tazobactam, and its use should not exceed 3 DDD/100 OBD. In addition, ABHR had a significant impact on reducing CRAb incidence when its use exceeded 2.6 L/100 OBD. Similar interpretations can be made for the identified antibiotics for the remaining hospitals (Table 1).

The identified thresholds can be translated into targets to inform hospital policies and antimicrobial stewardship (Table 2). Using the standard approach, the findings suggest that the use of certain antibiotics should be maintained below the identified thresholds in a number of hospitals. However, some hospitals will need to reduce their antibiotic use by certain percentages; for example, Khawlah Hospital (−20% piperacillin-tazobactam), As Sultan Qaboos Hospital (−14% fluoroquinolones), and Ibra Hospital (−27% piperacillin-tazobactam). The conservative approach recommends further reductions in the use of certain antibiotics (Table 2).

The identified thresholds for antibiotic and ABHR use and their effect on the incidence trends of CRAb in each hospital are shown in Figure 1. The association between identified variables and the incidence of CRAb, showing the estimated effect when use levels exceed their respective threshold value, is presented in Supplementary Materials Figures S1–S6. Graphs for the identified antibiotics, ABHR, and the incidence of CRAb on a monthly basis are presented in Supplementary Materials Figures S7–S12.

## 3. Discussion

This study found a non-linear association between the population use of specific antibiotics, ABHR, and CRAb incidence in six hospitals in Oman. Different antibiotics were associated with the incidence of CRAb, and different thresholds were identified across participating hospitals. Based on the identified thresholds, it was possible to provide hospital-specific quantitative targets for antibiotic stewardship improvement. Non-linear time series analysis methods have been applied to assess the relationship between antibiotic use and resistance in previously published research [10,11]

The assessment of the relationship between antibiotic use and the subsequent development of antimicrobial resistance necessitates the use of appropriate statistical methods [5,26,27]. Time series analysis, utilising linear methods, was applied to study the relationship between population antibiotic use and resistance [5,26]. However, theoretical and mathematical models suggest that non-linear relationships between antibiotic use and resistance are more frequent [9,28]. Therefore, non-linear analysis provides a more accurate estimate of the relationship between antibiotic use and resistance, enabling generations of thresholds that can be used to inform antimicrobial stewardship. Non-linear time-series analysis enables adjustment of the non-independence of serial observations inherent in antibiotic use and resistance time series and allows identification of temporality in associations and lag effects (delays needed to observe an effect).

The use of third-generation cephalosporins, piperacillin-tazobactam, aminoglycoside, and fluoroquinolones were associated with the incidence of CRAb in the study site hospitals. Evaluating the relevant resistance data obtained from the hospitals’ microbiology departments showed that CRAb isolates were resistant to the identified antibiotics (Table S2). Our findings are consistent with other published studies; nevertheless, the determined thresholds between identified antibiotics and CRAb incidence were different [10,11]. Thresholds may vary across populations depending on host, environment, and organism factors [10]. Although the present analysis was conducted on the same pathogen in one country, different thresholds were identified. These findings indicate the requirement to conduct thresholds analysis that is tailored to each hospital, taking into account differences in volumes of antibiotic use, microbiological and resistance profiles, and relevant healthcare systems and policies. In 2016, MOH launched the national antimicrobial guideline which includes therapeutics and surgical antimicrobial prophylaxis which guide the prescriber in the choice and duration of the antimicrobial agent [29,30]. Some of the hospitals are following international guidelines or hospital-specific guidelines.

The infection control department in each hospital is responsible for infection control precautions, isolations policy for CRAb, hand hygiene audits and round, and multidrug-resistant organism (MDRO) surveillance. The clinical microbiologists and clinical pharmacists are advising and monitoring the antibiotic choice, dose, and duration for the individual case [31]. Different antibiotic stewardship and infection control activities are shown in Table S3.

In the Royal Hospital, our findings showed that ABHR had an effect on reducing CRAb incidence up to a certain threshold, with no further impact measured beyond this (i.e., a ceiling effect). Similar findings were observed with certain antibiotics in another study in which it was proposed that inclusion of additional data may resolve it [10]. For Sur Hospital, ABHR had significant reduction on CRAb incidence when its use exceeded certain thresholds. The value of ABHR on reducing healthcare-acquired infections has been demonstrated in several studies [32,33,34].

Previous studies in Oman and the region reported frequent prescribing of third-generation cephalosporins, piperacillin/tazobactam, and fluoroquinolones [35,36,37,38,39,40]. In a recent point prevalence survey in the Middle East region, the authors demonstrated high prevalence rates of infections and high levels of resistance to antimicrobials [41]. Hospitals require guidance to inform their antimicrobial stewardship teams. Based on the finding of this study, the role of the AMS team begins by monitoring the specific antibiotic prescriptions in each hospital and updating the policy to reduce the consumption of antibiotics. Colistin and tigecycline are the drugs of choice in the sensitivity report used in the CRAb management. Therefore, with the limited available antibiotic choices and the increase in the use of antibiotics, certain strategies were applied e.g., pharmacokinetic and pharmacodynamic approaches [42,43]. Our study offers a new way to set targets for antibiotic use in hospitals by providing quantitative targets for antibiotic use that can inform antimicrobial stewardship [10]. Our findings recommended that the use of certain antibiotics should be maintained below the identified thresholds in a number of hospitals—that is to monitor its use with the aim of not exceeding the determined threshold. However, we provided targets for antibiotic use for some of the participating hospitals, for example, Khawlah Hospital (−20% piperacillin-tazobactam), As Sultan Qaboos Hospital (−14% fluoroquinolones), and Ibra Hospital (−27% piperacillin-tazobactam).

Many studies showed a reduction in the incidence of CRAb when implementing the infection control interventions, e.g., hand hygiene rounds, surveillance culture, contact precautions, environmental cleaning, and disinfection procedures. The infection control department has a big role in the hospital to reduce the HAIs and should extend the effort in applying the interventions to avoid an increase in the rate of CRAb [44,45].

This study identified antibiotics that are associated with CRAb and has provided quantitative targets for antimicrobial stewardship in the participating hospitals. Estimation of thresholds using both a standard and a conservative approach means that targets for control of resistance can be adjusted according to the hospital antimicrobial management team’s priorities using the lower limit of estimated threshold for stricter control of resistance. However, if there are challenges with implementation, then a standard approach can be adopted. As outlined by Lopez–Lozano and colleagues [10], and because antibiotic exposure may be important for individual patients, or cause changes in reservoirs of resistant pathogens in environments or hosts, it is important not to assume that all antibiotic use below thresholds is safe; thresholds should be used as a guide, rather than as strict limits, to achieve a balance between restriction of identified antibiotics and controlling resistance.

The present study used rigorous analysis methods, and included all hospitalized patients along with utilising routinely collected data; therefore selection and information bias are unlikely. However, the study has some limitations. It was not possible to adjust for potential changes in patient population and case mix. The addition of further explanatory variables can contribute to the improvement of presented models; for example, data on infection prevention and control activities (surface and medical equipment disinfection), and proxy measures for changes in patient population and case mix [46,47]. In this study, the latter data was not available. The findings of this study are related to the population of the Middle East, which may differ from other populations. In addition, it was not possible to capture patients that were admitted from the community with a known *Acinetobacter baumannii* infection. Further assessment, where sufficient observations are available, at the hospital’s unit level is needed as it may shape antibiotic use recommendations by providing specific thresholds (for example, intensive care unit versus other hospital units).

## 4. Methods

### 4.1. Study Design and Population

This was an ecological, multi-centre study that involved retrospective data collection. Six hospitals, from Oman, participated in this study: (i) Royal Hospital, (ii) Khawlah Hospital, (iii) As Sultan Qaboos Hospital, (iv) Nizwa Hospital, (v) Sur Hospital, and (vi) Ibra Hospital, and their general characteristics are summarised in the Supplementary Materials (Table S1). All inpatient (adult and paediatric) admissions were included in the study population. The time-series analysis requires at least 60 monthly observations (5 years) of antibiotic use and microbiological data [10,11], with all the hospitals being able to provide consistent monthly data for the explanatory and outcome variables for the entire study period (5-year dataset), i.e., January 2015 to December 2019, except for Sur Hospital (March 2015 to December 2019). For the purposes of this study, it was hypothesized that the use of carbapenems, fluoroquinolones, piperacillin/tazobactam, third-generation cephalosporins, and aminoglycosides could explain variations in the outcome of interest (incidence of CRAb cases). The aforementioned antibiotics were identified a priori on the basis of their resistance profiles (Table S2) and published evidence of their role as risk factors for driving the incidence of CRAb in hospitals [10,11]. In addition, ABHR was included in the analysis as one of the explanatory factors.

### 4.2. Microbiology and Pharmacy Data

Identification of isolates and antibiotic susceptibility tests were performed according to standard microbiological procedures and were in line with the Clinical and Laboratory Standards Institute (CLSI) guidelines [48,49]. Patients with an *A. baumannii* isolate from a clinical sample (i.e., blood, urine, fluid, wound, biopsy, cerebrospinal fluid (CSF), central venous pressure (CVP) line, catheter line, cannula line, ventriculoperitoneal (VP) shunt, sputum, and endotracheal tube (ET) secretion) that was resistant to meropenem were designated as a CRAb case. Infection control screening swabs were excluded. Duplication in CRAb cases were removed and any isolate identified within 30 days of a previous isolate from the same patient, with the same identification was considered as the same case. Data for antibiotic use quantities were obtained on a monthly basis and were converted into the number of DDD per 100 OBD. The DDD was calculated according to the classification of antimicrobials for systemic use (J01) in the WHO/ATC index [50]. The monthly consumption of ABHR (in liters) was also determined. All microbiological and pharmacy data was obtained from the Hospital Information Management System (Al Shifa 3 Plus). Data on the monthly incidence of CRAb cases and ABHR (L) was normalized per 100 OBD.

### 4.3. Statistical Analysis

The primary method employed to determine thresholds in antibiotic use levels that alter the trend in CRAb incidence rates was a flexible nonparametric regression modelling method, known as multivariate adaptive regression splines (MARS) [51]. This modelling strategy provides a generalized approach to recursive partitioning regression. A major assumption of a linear process is that the coefficients remain stable across all levels of the explanatory variables and, in the case of a time series, across all time periods. The MARS method is a very useful procedure when it is suspected that the model’s coefficients have different optimal values across different levels of the explanatory variables. It can detect and fit models where there are distinct breaks in the data, such as are found if there is a change in the underlying probability density function of the coefficients and where there are complicated variable interactions.

A more formal description of MARS is illustrated by first assuming a nonlinear model of the form

$$y=f\left(x_{1},\cdots ,x_{m}\right)+e$$

where subscript *m* signifies the number of explanatory variables $x_{1},\cdots ,x_{m}$ and *e* is random error. The MARS algorithm sets out to approximate the nonlinear function by

$$\hat{f}\left(\underline{X}\right)=\overset{s}{\underset{j=1}{\sum }}c_{j}K_{j}\left(\underline{X}\right),$$

where $\hat{f}\left(\underline{X}\right)$ is an additive function of the product basis functions ${\left\{K_{j}\left(\underline{X}\right)\right\}}_{j=1}^{s}$ associated with the *s* sub-regions ${\left\{R_{j}\right\}}_{j=1}^{s}$ and $c_{j}$ is the coefficient for the $j^{\mathrm{th}}$ product basis function. It is the algorithm that allows MARS to efficiently partition a dataset into what we can in simplest form consider a piece-wise regression model. For example, a MARS model may be partitioned around the breakpoint value of 100, such as

$$y=\alpha +\beta_{1}x+e\;\;\;\mathrm{for}\;x>100,\;\mathrm{and}$$

$$y=\alpha +\beta_{2}x+e\;\;\;\mathrm{for}\;x<100$$

which in MARS notational form would be

$$y=\alpha^{\prime }+c_{1}{\left(x-\tau^{*}\right)}_{+}+c_{2}{\left(\tau^{*}-x\right)}_{-}+e$$

where $\tau^{*}=100$ is the threshold value and (∙)_+_ and (∙)_−_ are the truncated spline functions which take on the value 0 if the expression inside (∙) is negative or its actual value if the expression inside (∙) is >0. Here $c_{1}\equiv \beta_{1}$ and $c_{2}\equiv \beta_{2}$.

Although MARS was the main method used, alternative methods were also adopted to confirm thresholds in explanatory variables and to enhance the MARS models and perform residual diagnostics. These were generalized additive models (GAM), segmented time-series models or threshold transfer function models, and segmented regression or piecewise regression models [52,53,54,55].

Initial analysis used GAM to explore curvilinear relationships and approximate threshold levels. The GAM estimation uses nonparametric fitting based on a scatter plot smoother to fit a polynomial relationship between two or more variables. The smoother summarizes the trend of the response variable as a function of the predictor variables by iteratively smoothing partial residuals in a process. Again, if we assume a nonlinear model of the form

$$y=f\left(x_{1},\cdots ,x_{m}\right)+e$$

where the individual explanatory variables, $x_{1},\cdots ,\;x_{m}\cdots$, and predictor variable, *y*, are one-dimensional vectors. A GAM model [52,55,56] can be written as

$$E(y|x_{1},x_{2},\ldots ,x_{k})=\alpha_{0}+\overset{k}{\underset{j=1}{\sum }}a_{j}\left(x_{j}\right)+e$$

where $\alpha_{j}$(∙) are smoothing functions standardized so that $E\propto_{j}\left(x_{j}\right)=0$.

After GAM was used to explore nonlinearities and approximate thresholds, MARS models were used to further explore thresholds and lagged relationships including lagged relationships in the response variable itself. Feasible models produced from the MARS modelling stage were then restated as threshold transfer function models where autoregressive moving average (ARMA) components can be added to the model to handle complex serial correlation, outlier detection and adjustment can be applied, and the model can be generally refined. A multiple-input transfer function takes on the general form

$$Y_{t}=C+\frac{\omega_{1}(B)}{\delta_{1}(B)}X_{1t}+\frac{\omega_{2}(B)}{\delta_{2}(B)}X_{2t}+\cdot \cdot \cdot +\frac{\omega_{m}(B)}{\delta_{m}(B)}X_{mt}+\frac{\theta (B)}{\phi (B)}a_{t}$$

where (*B*) is the backshift operator consistent with Box and Jenkins notation [53,57,58]. The rational transfer function for each input variable has the form

$$Y_{t}=C+\frac{\omega (B)}{\delta (B)}X_{t}+N_{t},N_{t}=\frac{\theta (B)}{\phi (B)}a_{t}$$

$\begin{array}{ll} \mathrm{where} & \omega \left(B\right)=(\omega_{0}+\omega_{1}B+\cdot \cdot \cdot +\omega_{h-1}B^{h-1})B^{b},\\ & \delta (B)=1-\delta_{1}B-\cdot \cdot \cdot -\delta_{r}B^{r},\\ & \phi (B)=1-\phi_{1}B-\cdot \cdot \cdot -\phi_{p}B^{p},\\ \mathrm{and} & \theta (B)=1-\theta_{1}B-\cdot \cdot \cdot -\theta_{q}B^{q}. \end{array}$

Additionally, the ϕ(B) and θ(B) terms are not limited as simple and may be in multiplicative form. In the above model, $N_{t}$ is referred to as the disturbance, and $a_{t}$ is a sequence of random shocks that are independently and identically distributed. The shocks are assumed to be independent of $X_{t}$ and its associated lags.

In addition, confirmatory analysis was conducted on the identified thresholds by using segmented regression and the interval for the breakpoint parameter was derived from a gradient estimation approach [55].

Patient treatments per month were estimated to translate the identified thresholds into recommendations for informing population-specific antimicrobial stewardship policies. The identified threshold (DDD/100 OBD) was multiplied by the size of the population (OBD), and then divided by 7 DDD (i.e., average patient treatment). Two approaches were followed to present estimates for informing antimicrobial stewardship policies: a standard approach, based on using the identified threshold and a conservative approach, based on using the lower limit as the threshold. All analysis was carried out by using the SCA Statistical System version 8.2 (Scientific Computing Associates Corp., River Forest, IL, USA) and R software (R Foundation for Statistical Computing, Vienna, Austria).

## 5. Conclusions

In conclusion, we measured thresholds in the association between third-generation cephalosporins, piperacillin-tazobactam, aminoglycoside, and fluoroquinolones and the CRAb incidence in six hospitals. These hospitals had different sizes, specialties, volumes of antibiotics, and CRAb incidence rates. Identification of different thresholds, due to differences between the hospitals in these factors emphasises the need to carry out tailored analysis, based on modelling data in each hospital. The identified thresholds can be used to set targets for hospital antibiotic stewardship. The complete restriction of antibiotics in clinical practice is challenging and would likely lead to compensatory use of different antibiotics (squeezing the balloon) bringing other resistance issues [59]. By providing thresholds it should be possible to establish a balance between antibiotic use and CRAb incidence, while maintaining diversity of prescribing. Further studies are needed to assess the feasibility of identifying and implementing thresholds into routine clinical practice and to assess their value in informing antimicrobial stewardship and controlling resistance rates in hospitals.

## Figures and Tables

**Figure 1:**
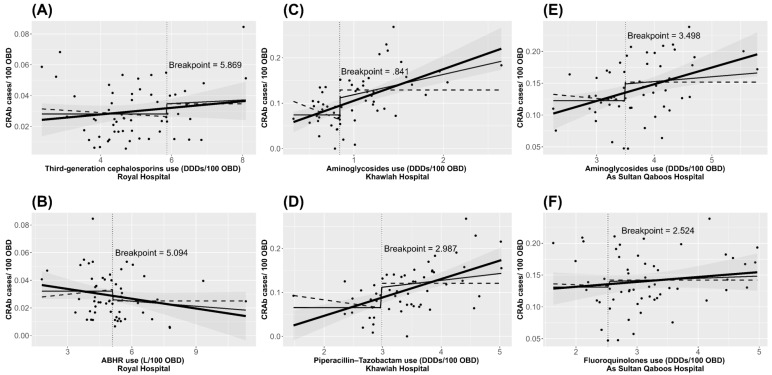

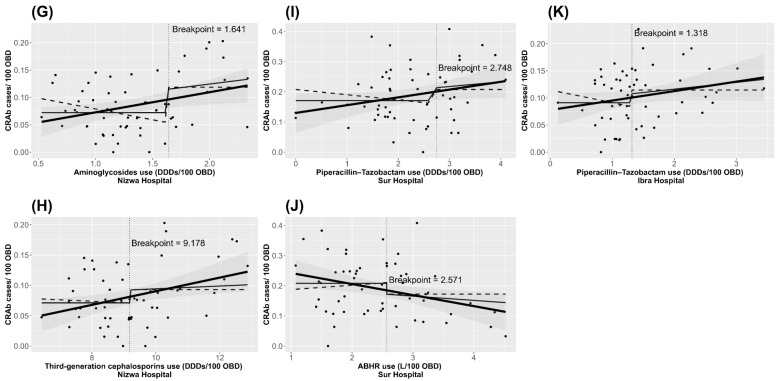
Charts illustrating the identified thresholds for antibiotic (**A**,**C**–**I**,**K**) and ABHR (**B**,**J**) use and their effect on CRAb incidence trends, January 2015 to December 2019 in hospitals in Oman. The thick black line is the fitted linear regression line of the unsegmented data. The thin black line is the fitted linear regression line focusing on x > threshold and the dashed black line focuses on x < threshold.

**Table 1:** Results of multivariable non-linear time-series analyses, January 2015 to December 2019.

Hospitals	Terms	Median Use (IQ Range) *	Lag (Months)	Threshold (95% Confidence Limit)	Relationship to Threshold	Regression Coefficient (95% CI)	*p* Value
a. Royal Hospital (R^2^ = 38.2)	Constant	N/A	N/A	N/A	N/A	0.024 (0.020 to 0.029)	<0.0001
	Third-generation cephalosporins	4.82 (4.24–5.85)	1	5.87 (5.39 to 7.64)	Above	0.017 (0.010 to 0.024)	<0.0001
	Alcohol-based hand rub	5.03 (4.20–5.50)	3	5.09 (2.20 to 5.11)	Below	0.008 (0.003 to 0.013)	0.0025
b. Khawlah Hospital (R^2^ = 47.58)	Constant	N/A	N/A	N/A	N/A	0.074 (0.057 to 0.091)	<0.0001
	Piperacillin-tazobactam	3.40 (2.87–3.83)	2	2.99 (2.35 to 3.86)	Above	0.029 (0.010 to 0.047)	0.0039
	Aminoglycosides	0.92 (0.66–1.22)	1	0.84 (0.81 to 1.40)	Above	0.053 (0.024 to 0.081)	0.0005
	Autoregressive	N/A	1	N/A	N/A	0.328 (0.076 to 0.581)	0.0154
c. As Sultan Qaboos Hospital (R^2^ = 30.04)	Constant	N/A	N/A	N/A	N/A	0.109 (0.092 to 0.126)	<0.0001
	Aminoglycosides	3.54 (3.21–4.13)	4	3.50 (3.44 to 5.46)	Above	0.046 (0.026 to 0.067)	<0.0001
	Fluoroquinolones	2.98 (2.57–3.41)	4	2.52 (2.00 to 2.72)	Above	0.026 (0.010 to 0.041)	0.0022
d. Nizwa Hospital (R^2^ = 24.50)	Constant	N/A	N/A	N/A	N/A	0.068 (0.055 to 0.082)	<0.0001
	Third-generation cephalosporins	9.05 (8.08–10.02)	4	9.18 (9.15 to 12.52)	Above	0.014 (0.002 to 0.026)	0.0246
	Aminoglycosides	1.27 (1.00–1.63)	2	1.64 (1.32 to 1.70)	Above	0.079 (0.009 to 0.148)	0.0339
e. Sur Hospital (R^2^ = 42.9)	Constant	N/A	N/A	N/A	N/A	0.204 (0.179 to 0.228)	<0.0001
	Piperacillin-tazobactam	2.29 (1.76–2.99)	3	2.75 (1.83 to 3.77)	Above	0.070 (0.006 to 0.135)	0.0387
	Alcohol-based hand rub	2.51 (1.87–3.08)	2	2.57 (2.15 to 3.06)	Above	−0.079 (−0.118 to −0.041)	0.0001
f. Ibra Hospital (R^2^ = 9.2)	Constant	N/A	N/A	N/A	N/A	0.085 (0.066 to 0.104)	<0.0001
	Piperacillin-tazobactam	1.30 (1.04–1.79)	3	1.32 (1.12 to 2.98)	Above	0.016 (0.003 to 0.028)	0.0219

* Defined Daily Dose (DDD) per 100 occupied bed-days (OBD).

**Table 2:** Translation of thresholds identified in non-linear models into population-specific antimicrobial stewardship policy suggestions.

Hospitals	Antibiotic	Patient Treatments per Month			
		Maximum Suggested by Threshold (Lower and Upper Bound)	Average Use in Last 12 Months of Study	Suggested Reduction in Use (%)	
				Standard	Conservative
a. Royal Hospital	Third-generation cephalosporins	139 (127–181)	105	Maintain below threshold	Maintain below threshold
b. Khawlah Hospital	Piperacillin-tazobactam	44 (35–57)	55	11 (20)	20 (36)
	Aminoglycoside	12 (12–21)	11	Maintain below threshold	Maintain below threshold
c. As Sultan Qaboos Hospital	Aminoglycoside	52 (51–84)	49	Maintain below threshold	Maintain below threshold
	Fluoroquinolones	38 (30–41)	44	6 (14)	14 (32)
d. Nizwa Hospital	Third-generation cephalosporins	85 (84–115)	74	Maintain below threshold	Maintain below threshold
	Aminoglycoside	15 (12–16)	9	Maintain below threshold	Maintain below threshold
e. Sur Hospital	Piperacillin-tazobactam	13 (9–15)	12	Maintain below threshold	3 (25)
f. Ibra Hospital	Piperacillin-tazobactam	8 (7–19)	11	3 (27)	4 (36)

## Data Availability

Collected data for this study are published in this article.

## Supplementary Materials

The following are available online at https://www.mdpi.com/article/10.3390/antibiotics11060775/s1. Table S1: General characteristics of participating hospitals from Oman; Table S2: Rates of non-susceptibility to certain selecting antibiotics in carbapenem-resistant *Acinetobacter baumannii* isolates from participating hospitals in Oman; Table S3: General characteristics of antibiotic stewardship and infection control in participating hospitals from Oman; Figure S1: Contribution charts illustrate the relationship between third-generation cephalosporins, alcohol-based hand rub and the incidence of CRAb, showing the estimated effect when use levels exceed their respective threshold value, Royal Hospital; Figure S2: Contribution charts illustrate the relationship between piperacillin-tazobactam, aminoglycoside and the incidence of CRAb, showing the estimated effect when use levels exceed their respective threshold value, Khawlah Hospital; Figure S3: Contribution charts illustrate the relationship between aminoglycoside, fluoroquinolones and the incidence of CRAb, showing the estimated effect when use levels exceed their respective threshold value, As Sultan Qaboos Hospital; Figure S4: Contribution charts illustrate the relationship between third-generation cephalosporins, aminoglycoside and the incidence of CRAb, showing the estimated effect when use levels exceed their respective threshold value, Nizwa Hospital; Figure S5: Contribution charts illustrate the relationship between piperacillin-tazobactam, alcohol-based hand rub and the incidence of CRAb, showing the estimated effect when use levels exceed their respective threshold value, Sur Hospital; Figure S6: Contribution charts illustrate the relationship between piperacillin-tazobactam and the incidence of CRAb, showing the estimated effect when use levels exceed their respective threshold value, Ibra Hospital; Figure S7: Monthly incidence of carbapenem-resistant *Acinetobacter baumannii* (CRAb) versus third-generation cephalosporins use and use of alcohol-based hand rub, Royal Hospital, January 2015–December 2019; Figure S8: Monthly incidence of carbapenem-resistant *Acinetobacter baumannii* (CRAb) versus use of aminoglycosides and piperacillin–tazobactam, Khawlah Hospital, January 2015-December 2019; Figure S9: Monthly incidence of carbapenem-resistant *Acinetobacter baumannii* (CRAb) versus use of aminoglycosides and fluoroquinolones, As Sultan Qaboos Hospital, January 2015-December 2019; Figure S10: Monthly incidence of carbapenem-resistant *Acinetobacter baumannii* (CRAb) versus third-generation cephalosporins and aminoglycosides use, Nizwa Hospital, January 2015-December 2019; Figure S11: Monthly incidence of carbapenem-resistant *Acinetobacter baumannii* (CRAb) versus piperacillin-tazobactam use and alcohol-based hand rub use, Sur Hospital, March 2015-December 2019; Figure S12: Monthly incidence of carbapenem-resistant *Acinetobacter baumannii* (CRAb) versus piperacillin-tazobactam use, Ibra Hospital, January 2015–December 2019.

## Abbreviations

ABHR	Alcohol-Based Hand Rub
AMR	Antimicrobial Resistance
AMS	Antimicrobial Stewardship
ARMA	Autoregressive Moving Average
ATC	Anatomical Therapeutic Classification
CLSI	Clinical and Laboratory Standards Institute
CRAb	Carbapenem-Resistant *Acinetobacter Baumannii*
CRE	Carbapenem-resistant Enterobacteriaceae
CSF	Cerebrospinal fluid
CVP	Central venous pressure
DDD	Defined Daily Dose
ET	Endotracheal tube
GAM	Generalized Additive Models
HAIs	Health-care-associated infections
J01	Antibacterials for systemic use
L	Liter
MARS	Multivariate Adaptive Regression Splines
MDRAb	Multidrug-resistant *Acinetobacter baumannii*
MDRO	Multidrug-resistant organism
MOH	Ministry of Health
OBD	Occupied Bed-Days
OMASS	Oman Antimicrobial Surveillance System
SCA	Scientific Computing Associates
VP	Ventriculoperitoneal
WHO	World Health Organization

## References

[1] Aldeyab M., López-Lozano J.M., Gould I.M., Babar Z.U.D. (2020). Global antibiotics use and resistance. Global Pharmaceutical Policy.

[2] O’Neill J. (2014). Antimicrobial Resistance: Tackling a Crisis for the Health and Wealth of Nations. The Review on Antimicrobial Resistance. https://amr-review.org/Publications.html.

[3] Antimicrobial Resistance Collaborators (2022). Global burden of bacterial antimicrobial resistance in 2019: A systematic analysis. Lancet.

[4] Hecker M.T., Aron D.C., Patel N.P., Lehmann M.K., Donskey C.J. (2003). Unnecessary use of antimicrobials in hospitalized patients: Current patterns of misuse with an emphasis on the antianaerobic spectrum of activity. Arch. Intern. Med..

[5] Jirjees F.J., Al-Obaidi H.J., Sartaj M., Conlon-Bingham G., Farren D., Scott M.G., Gould I.M., López-Lozano J.M., Aldeyab M.A. (2020). Antibiotic use and resistance in hospitals: Time-series analysis strategy for determining and prioritising interventions. Hosp Pharm Eur..

[6] Levy S.B., Marshall B. (2004). Antibacterial resistance worldwide: Causes, challenges and responses. Nat. Med..

[7] Davies J., Davies D. (2010). Origins and evolution of antibiotic resistance. Microbiol. Mol. Biol. Rev..

[8] Davey P., Brown E., Charani E., Fenelon L., Gould I.M., Holmes A., Ramsay C.R., Wiffen P.J., Wilcox M. (2017). Interventions to improve antibiotic prescribing practices for hospital inpatients. Cochrane Database Syst. Rev..

[9] Levy S.B. (1994). Balancing the drug-resistance equation. Trends Microbiol..

[10] López-Lozano J.M., Lawes T., Nebot C., Beyaert A., Bertrand X., Hocquet D., Aldeyab M., Scott M., Conlon-Bingham G., Farren D. (2019). A nonlinear time-series analysis approach to identify thresholds in associations between population antibiotic use and rates of resistance. Nat. Microbiol..

[11] Hayajneh W.A., Al-Azzam S., Yusef D., Lattyak W.J., Lattyak E.A., Gould I., López-Lozano J.M., Conway B.R., Conlon-Bingham G., Aldeyab M.A. (2021). Identification of thresholds in relationships between specific antibiotic use and carbapenem-resistant Acinetobacter baumannii (CRAb) incidence rates in hospitalized patients in Jordan. J. Antimicrob. Chemother..

[12] Yusef D., Hayajneh W.A., Bani Issa A., Haddad R., Al-Azzam S., Lattyak E.A., Lattyak W.J., Gould I., Conway B.R., Bond S. (2021). Impact of an antimicrobial stewardship programme on reducing broad-spectrum antibiotic use and its effect on carbapenem-resistant Acinetobacter baumannii (CRAb) in hospitals in Jordan. J. Antimicrob. Chemother..

[13] Oman: Antimicrobial Resistance (AMR) National Action Plan. https://cdn.who.int/media/docs/default-source/antimicrobial-resistance/amr-spc-npm/nap-library/oman_national-action-plan-on-amr.pdf?sfvrsn=7f35d99_1&download=true.

[14] Oman Antimicrobial Resistance Surveillance System (OMASS) (2018). The First National Annual Antimicrobial Resistance Report (2017).

[15] Oman Antimicrobial Resistance Surveillance System (OMASS) (2019). The Annual Antimicrobial Resistance Report (2018).

[16] Balkhair A., Al-Muharrmi Z., Al’Adawi B., Al Busaidi I., Taher H.B., Al-Siyabi T., Al Amin M., Hassan K.S. (2019). Prevalence and 30-day all-cause mortality of carbapenem-and colistin-resistant bacteraemia caused by Acinetobacter baumannii, Pseudomonas aeruginosa, and Klebsiella pneumoniae: Description of a decade-long trend. Int. J. Infect. Dis..

[17] WHO (2009). WHO guidelines on hand hygiene in health care. First Global Patient Safety Challenge—Clean Ecare Is Safer Care.

[18] Kampf G., Löffler H., Gastmeier P. (2009). Hand hygiene for the prevention of nosocomial infections. Dtsch. Arztebl. Int..

[19] Lotfinejad N., Peters A., Tartari E., Fankhauser-Rodriguez C., Pires D., Pittet D. (2021). Hand hygiene in health care: 20 years of ongoing advances and perspectives. Lancet Infect. Dis..

[20] Lawes T., López-Lozano J.-M., Nebot C.A., Macartney G., Subbarao-Sharma R., Dare C., Wares K.D., Gould I.M. (2015). Effects of national antibiotic stewardship and infection control strategies on hospital-associated and community-associated meticillin-resistant Staphylococcus aureus infections across a region of Scotland: A non-linear time-series study. Lancet Infect. Dis..

[21] Lawes T., López-Lozano J.-M., Nebot C., Macartney G., Subbarao-Sharma R., Dare C.R.J., Edwards G.F.S., Gould I.M. (2015). Turning the tide or riding the waves? Impacts of antibiotic stewardship and infection control on MRSA strain dynamics in a Scottish region over 16 years: Non-linear time series analysis. BMJ Open.

[22] Lawes T., López-Lozano J.-M., Nebot C.A., Macartney G., Subbarao-Sharma R., Wares K.D., Sinclair C., Gould I.M. (2017). Effect of a national 4C antibiotic stewardship intervention on the clinical and molecular epidemiology of Clostridium difficile infections in a region of Scotland: A non-linear time-series analysis. Lancet Infect. Dis..

[23] Laxminarayan R., Duse A., Wattal C., Zaidi A.K.M., Wertheim H.F.L., Sumpradit N., Vlieghe E., Hara G.L., Gould I.M., Goossens H. (2013). Antibiotic resistance—the need for global solutions. Lancet Infect. Dis..

[24] Isler B., Doi Y., Bonomo R.A., Paterson D.L. (2018). New treatment options against carbapenem-resistant Acinetobacter baumannii infections. Antimicrob. Agents Chemother..

[25] Wong D., Nielsen T.B., Bonomo R.A., Pantapalangkoor P., Luna B., Spellberg B. (2017). Clinical and pathophysiological overview of Acinetobacter infections: A century of challenges. Clin. Microbiol. Rev..

[26] López-Lozano J.M., Monnet D.L., Yagüe A., Burgos A., Gonzalo N., Campillos P., Saez M. (2000). Modelling and forecasting antimicrobial resistance and its dynamic relationship to antimicrobial use: A time series analysis. Int. J. Antimicrob. Agents.

[27] Shardell M., Harris A.D., El-Kamary S.S., Furuno J.P., Miller R.R., Perencevich E.N. (2007). Statistical analysis and application of quasi experiments to antimicrobial resistance intervention studies. Clin. Infect. Dis..

[28] Austin D.J., Kristinsson K.G., Anderson R.M. (1999). The relationship between the volume of antimicrobial consumption in human communities and the frequency of resistance. Proc. Natl. Acad. Sci. USA.

[29] National Surgical Antimicrobial Prophylaxis Guidelines. https://moh.gov.om/documents/236878/0/national+surgical+antimicrobial+prophylaxis/dd57462f-2f8b-47c6-b78b-f2821ad17fc9.

[30] National Antimicrobial Guidelines. https://www.moh.gov.om/documents/236878/0/national+antimicrobial+guidelines/c82511c5-63e9-4205-8f49-0f60df4d7aa4.

[31] MoH Code of Practice for Infection Prevention and Control. https://www.moh.gov.om/documents/236878/0/MOH+Code+Practice/4e55e63c-a1dc-4a2d-b813-37ec6915dc60.

[32] Allegranzi B., Pittet D. (2009). Role of hand hygiene in healthcare-associated infection prevention. J. Hosp. Infect..

[33] Barrera L., Zingg W., Mendez F., Pittet D. (2011). Effectiveness of a hand hygiene promotion strategy using alcohol-based handrub in 6 intensive care units in Colombia. Am. J. Infect. Control.

[34] Kingston L., O’Connell N.H., Dunne C.P. (2016). Hand hygiene-related clinical trials reported since 2010: A systematic review. J. Hosp. Infect..

[35] Haseeb A., Faidah H.S., Algethamy M., Alghamdi S., Alhazmi G.A., Alshomrani A.O., Alqethami B.R., Alotibi H.S., Almutiri M.Z., Almuqati K.S. (2021). Antimicrobial Usage and Resistance in Makkah Region Hospitals: A Regional Point Prevalence Survey of Public Hospitals. Int. J. Environ. Res. Public Health.

[36] Al-Maliky G.R., Al-Ward M.M., Taqi A., Balkhair A., Al-Zakwani I. (2018). Evaluation of antibiotic prescribing for adult inpatients at Sultan Qaboos University Hospital, Sultanate of Oman. Eur. J. Hosp. Pharm..

[37] Al-Yamani A., Khamis F., Al-Zakwani I., Al-Noomani H., Al-Noomani J., Al-Abri S. (2016). Patterns of Antimicrobial Prescribing in a Tertiary Care Hospital in Oman. Oman Med. J..

[38] Mahmood R.K., Gillani S.W., Saeed M.W., Hafeez M.U., Gulam S.M. (2020). Systematic Review: Study of the Prescribing Pattern of Antibiotics in Outpatients and Emergency Departments in the Gulf Region. Front. Pharmacol..

[39] Elhajji F.D., Al-Taani G.M., Anani L., Al-Masri S., Abdalaziz H., Qabba’H S.H., Al Bawab A.Q., Scott M., Farren D., Gilmore F. (2018). Comparative point prevalence survey of antimicrobial consumption between a hospital in Northern Ireland and a hospital in Jordan. BMC Health Serv. Res..

[40] Al Matar M., Enani M., Binsaleh G., Roushdy H., Alokaili D., Al Bannai A., Khidir Y., Al-Abdely H. (2019). Point prevalence survey of antibiotic use in 26 Saudi hospitals in 2016. J. Infect. Public Health.

[41] Alothman A., Al Thaqafi A., Al Ansary A., Zikri A., Fayed A., Khamis F., Al Salman J., Al Dabal L., Khalife N., AlMusawi T. (2020). Prevalence of infections and antimicrobial use in the acute-care hospital setting in the Middle East: Results from the first point-prevalence survey in the region. Int. J. Infect. Dis..

[42] Livermore D.M., Hill R.L., Thomson H., Charlett A., Turton J., Pike R., Patel B.C., Manuel R., Gillespie S., Balakrishnan I. (2010). Antimicrobial treatment and clinical outcome for infections with carbapenem- and multiply-resistant Acinetobacter baumannii around London. Int. J. Antimicrob. Agents.

[43] Garnacho-Montero J., Amaya-Villar R., Ferrándiz-Millón C., Díaz-Martín A., López-Sánchez J.M., Gutiérrez-Pizarraya A. (2015). Optimum treatment strategies for carbapenem-resistantAcinetobacter baumanniibacteremia. Expert Rev. Anti-Infect. Ther..

[44] Hong J., Jang O.J., Bak M.H., Baek E.H., Park K.-H., Hong S.I., Cho O.-H., Bae I.-G. (2018). Management of carbapenem-resistant Acinetobacter baumannii epidemic in an intensive care unit using multifaceted intervention strategy. Korean J. Intern. Med..

[45] Meschiari M., Lòpez-Lozano J.-M., Di Pilato V., Gimenez-Esparza C., Vecchi E., Bacca E., Orlando G., Franceschini E., Sarti M., Pecorari M. (2021). A five-component infection control bundle to permanently eliminate a carbapenem-resistant Acinetobacter baumannii spreading in an intensive care unit. Antimicrob. Resist. Infect. Control.

[46] Aldeyab M.A., McELNAY J.C., Scott M.G., Elhajji F.W.D., Kearney M.P. (2014). Hospital antibiotic use and its relationship to age-adjusted comorbidity and alcohol-based hand rub consumption. Epidemiol. Infect..

[47] Aldeyab M.A., McElnay J.C., Scott M., Lattyak W.J., Elhajji F.D., Aldiab M.A., Magee F.A., Conlon G., Kearney M.P. (2014). A modified method for measuring antibiotic use in healthcare settings: Implications for antibiotic stewardship and benchmarking. J. Antimicrob. Chemother..

[48] Magiorakos A.-P., Srinivasan A., Carey R.B., Carmeli Y., Falagas M.E., Giske C.G., Harbarth S., Hindler J.F., Kahlmeter G., Olsson-Liljequist B. (2012). Multidrug-resistant, extensively drug-resistant and pandrug-resistant bacteria: An international expert proposal for interim standard definitions for acquired resistance. Clin. Microbiol. Infect..

[49] CLSI (2016). Performance Standards for Antimicrobial Susceptibility Testing—Twenty-Sixth Edition: M100.

[50] WHO Collaborating Centre for Drug Statistics Methodology (2021). Guidelines for ATC Classification and DDD Assignment, 2022. Oslo. https://www.whocc.no/filearchive/publications/2022guidelinesweb.pdf.

[51] Friedman J. (1991). Multivariate adaptive regression splines. Ann. Statist..

[52] Hastie T., Tibshirani R. (1990). Generalized Additive Models.

[53] Liu L.-M. (2009). Time Series Analysis and Forecasting.

[54] Neter J., Wasserman W., Kutner M.H. (1990). Applied Linear Statistical Models.

[55] Muggeo V.M.R. (2017). Interval estimation for the breakpoint in segmented regression: A smoothed score-based approach. Austral. N. Z. J. Stat..

[56] Faraway J. (2006). Extending the Linear Model with R.

[57] Box G.E.P., Jenkins G.M. (1976). Time Series Analysis: Forecasting and Control.

[58] Box G.E.P., Jenkins G.M., Reinsel G.C. (1994). Time Series Analysis: Forecasting and Control.

[59] Conlon-Bingham G.M., Aldeyab M., Scott M., Kearney M.P., Farren D., Gilmore F., McElnay J. (2019). Effects of antibiotic cycling policy on incidence of healthcare-associated MRSA and Clostridioides difficile infection in secondary healthcare settings. Emerg. Infect. Dis..

