# Supplementary material for: Identifying Targets for Antibiotic Use for the Management of Carbapenem-Resistant Acinetobacter baumannii (CRAb) in Hospitals—A Multi-Centre Nonlinear Time-Series Study
Source: Antibiotics (Basel). 2022 Jun 7;11(6):775. doi: 10.3390/antibiotics11060775 (PMC9220031; doi:10.3390/antibiotics11060775)
Supplement: Supplementary file 1 [file antibiotics-11-00775-s001.zip › antibiotics-1747549-supplementary.pdf]

Supplementary Materials

Table S1. General Characteristics of participating hospitals from Oman

Specialty		Royal Hospital	Khawlah Hospital	As Sultan Qaboos Hospital	Nizwa Hospital	Sur Hospital	Ibra Hospital
**Medicine**	General Medicine	114		66	60	21	47
	Neurology		24				
	Haematology	16					
	Oncology	50					
	Cardiology	74		52		7	
	Nephrology	14				5	
	Psychiatry			11			
**Surgery**	General Surgery	64	35	39	31	24	24
	Hand Surgery		14				
	Plastic surgery		72	11			
	Burns Unit		12		6		4
	Orthopaedic Surgery		106	28	18	15	15
	Paediatric Surgery	24	36	6	7		
	Neuro Surgery		50	8			
	Urology	24					
	Cardio-thoracic	38					
	Ophthalmology			9	9	15	9
	ENT			9	11	15	9
**Paediatrics**	SCBU	36	47	38	21	18	18
	Paediatrics	76		71	44	29	16
	ICU	8		4	4	4	
	Paediatrics Isolation						1
Gynaecology & Obstetrics		140	83	88	60	55	28
ICU		14	30	18	8	8	4
CCU		8		4	8	7	3
VIP Ward		12	30	47	10		6
Isolation Room		12		6	8	13	7
Special Nursing Ward		15					
**Total number of beds**		**739**	**539**	**515**	**305**	**236**	**191**
**Mean monthly bed days**		**16540.3**	**10340.7**	**10469.7**	**6444.2**	**3456.3**	**4493.5**

ENT: Ear, Nose and Throat. SCBU: Special Care Baby Unit. ICU: Intensive Care Unit. CCU: Cardiac Care Unit. VIP: Very Important Person

Table S2. Rates of non-susceptibility to certain selecting antibiotics in carbapenem-resistant *Acinetobacter baumannii* isolates from participating hospitals in Oman

		Royal Hospital CRAb isolates (*n*= 295)		Khawlah Hospital CRAb isolates (*n*=646)		As Sultan Qaboos CRAb isolates (*n*= 874)	
**ATC antibiotic sub-group**	**Antibiotic**	**Tested**	**I+R (%)**	**Tested**	**I+R (%)**	**Tested**	**I+R (%)**
Third generation cephalosporin	Ceftazidime	294	98.3%	645	97.5%	872	99.5%
	Ceftriaxone	290	98.6%	55	100.0%	870	99.9%
Fluoroquinolones	Ciprofloxacin	295	98.6%	643	97.0%	872	99.7%
Aminoglycoside	Amikacin	295	89.5%	598	88.1%	847	96.0%
	Gentamicin	295	96.9%	645	89.8%	873	97.9%
Combined penicillin and β-lactamase inhibitor	Piperacillin+Tazobactam	295	99.0%	635	98.0%	872	99.8%
Polymyxins	Colistin	287	1.0%	355	1.7%	749	1.7%
Carbapenam	Meropenem	295	100.0%	646	100.0%	874	100.0%

		Nizwa Hospital CRAb isolates (*n*= 336)		Sur Hospital CRAb isolates (*n*= 387)		Ibra Hospital CRAb isolates (*n*= 282)	
**ATC antibiotic sub-group**	**Antibiotic**	**Tested**	**I+R (%)**	**Tested**	**I+R (%)**	**Tested**	**I+R (%)**
Third generation cephalosporin	Ceftazidime	336	99%	387	95%	275	99.3%
	Ceftriaxone	306	100%	4	100%	240	100.0%
Fluoroquinolones	Ciprofloxacin	336	100%	385	84%	267	99.3%
Aminoglycoside	Amikacin	335	94%	386	81%	277	52.7%
	Gentamicin	336	97%	384	79%	277	96.4%
Combined penicillin and β-lactamase inhibitor	Piperacillin+Tazobactam	336	99%	349	91%	276	98.9%
Polymyxins	Colistin	325	1%	123	15%	253	1.2%
Carbapenam	Meropenem	336	100%	387	100%	282	100.0%

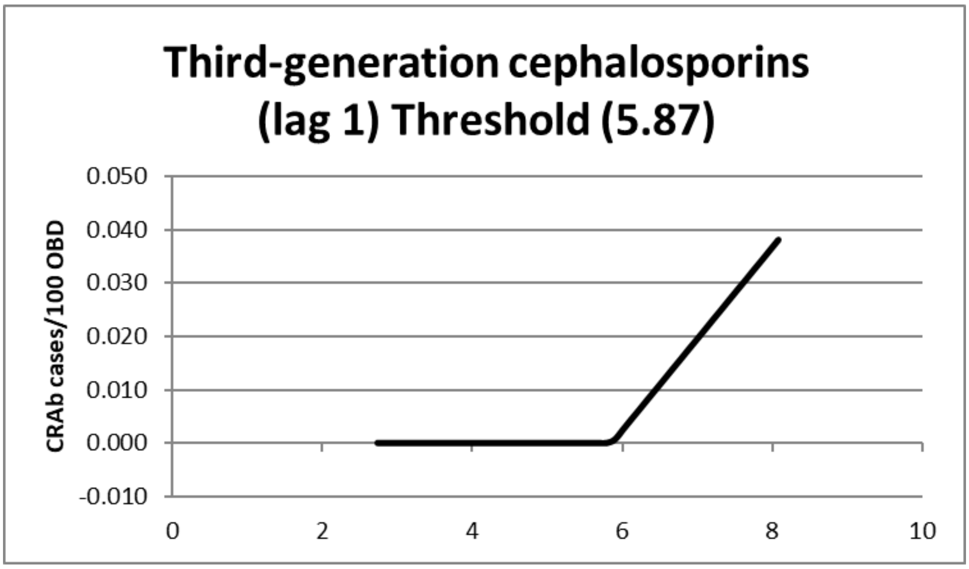

(A)

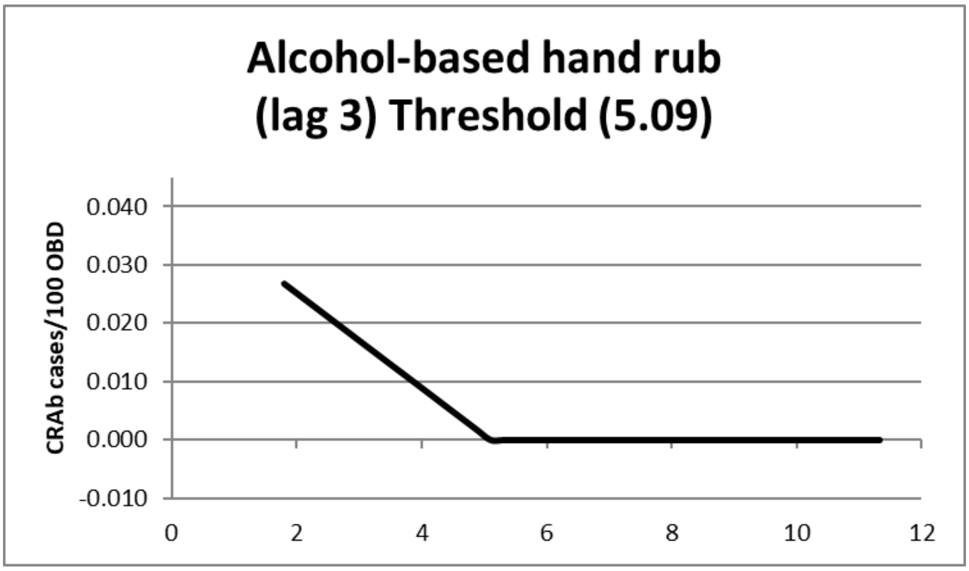

(B)

Figure S1. Contribution charts illustrate the relationship between third-generation cephalosporins (**A**), alcohol-based hand rub (**B**) and the incidence of CRAb, showing the estimated effect when use levels exceed their respective threshold value, Royal Hospital.

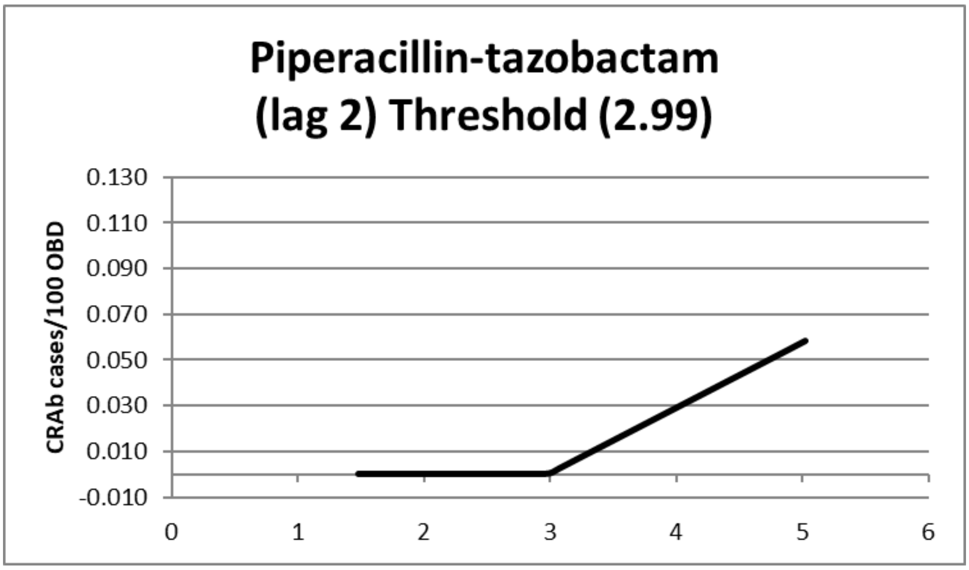

(A)

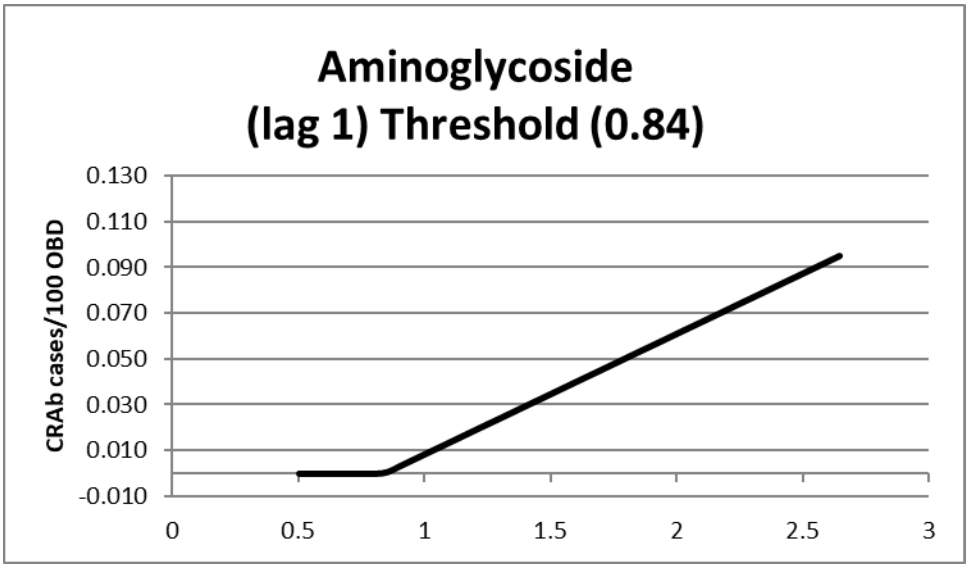

(B)

Figure S2. Contribution charts illustrate the relationship between piperacillin-tazobactam (**A**), aminoglycoside (**B**) and the incidence of CRAb, showing the estimated effect when use levels exceed their respective threshold value, Khawlah Hospital.

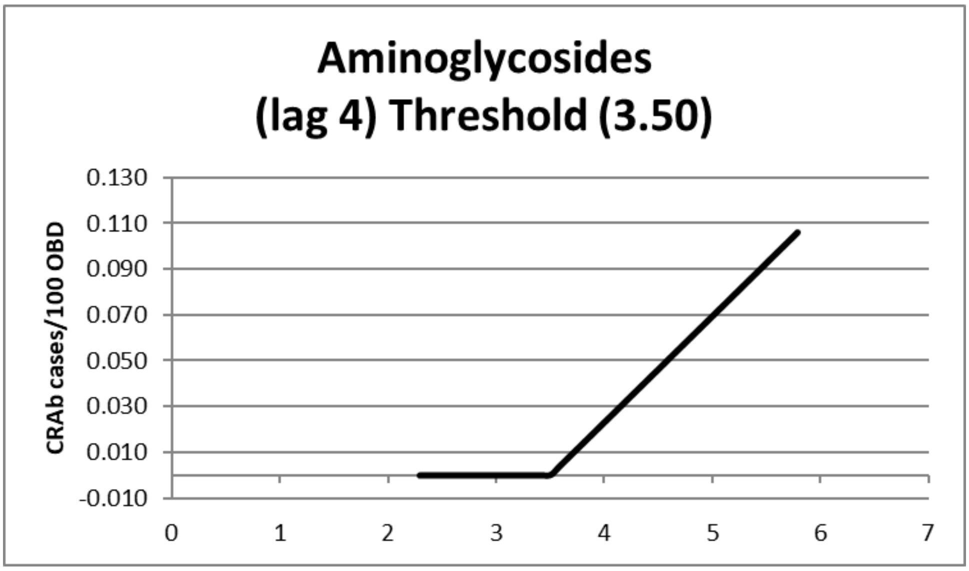

(A)

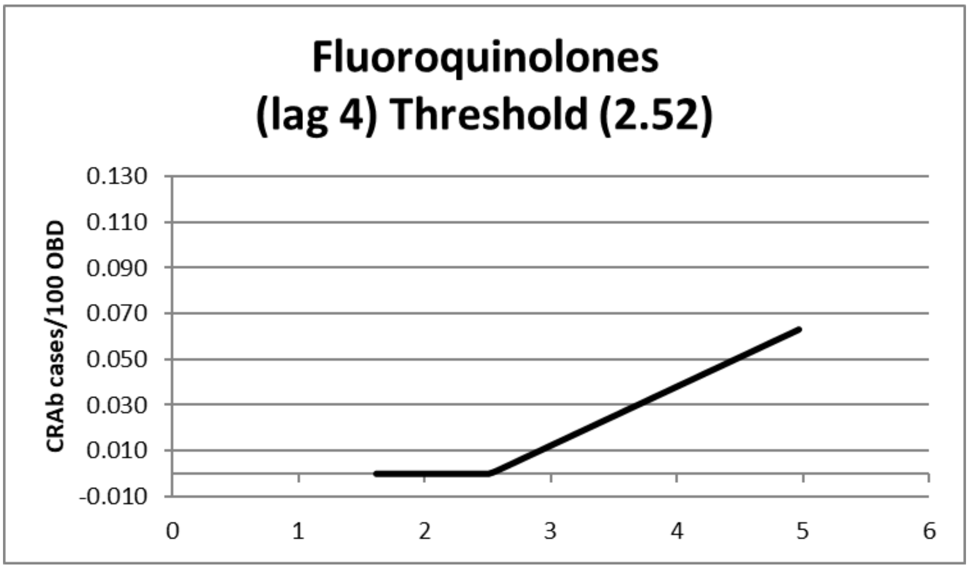

(B)

Figure S3. Contribution charts illustrate the relationship between aminoglycoside (**A**), fluoroquinolones (**B**), and the incidence of CRAb, showing the estimated effect when use levels exceed their respective threshold value, As Sultan Qaboos Hospital.

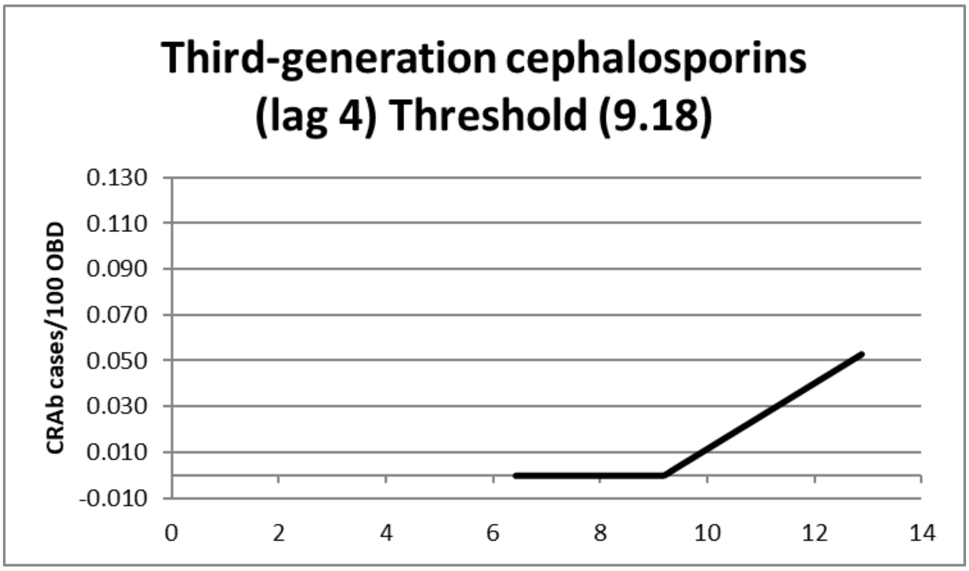

(A)

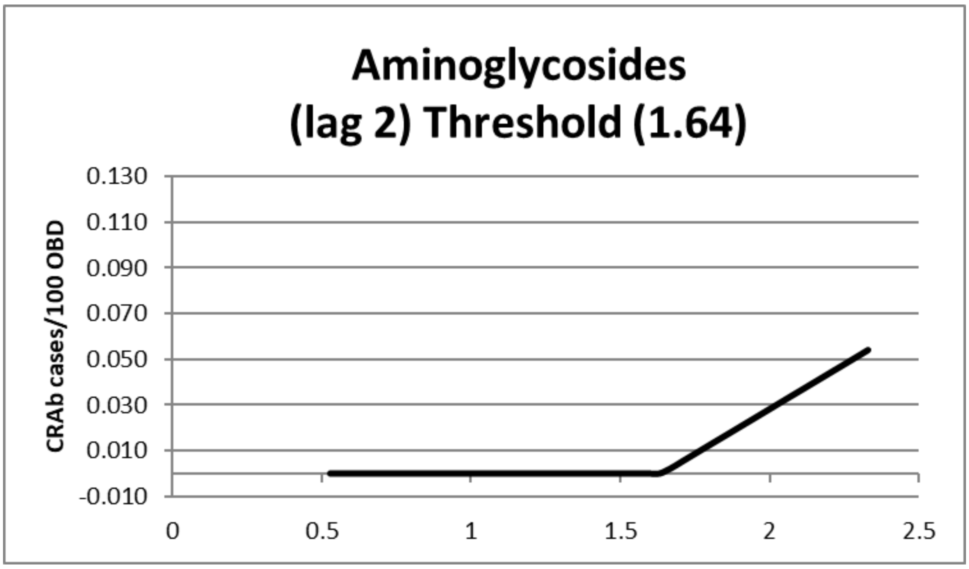

(B)

Figure S4. Contribution charts illustrate the relationship between third-generation cephalosporins (**A**), aminoglycoside (**B**), and the incidence of CRAb, showing the estimated effect when use levels exceed their respective threshold value, Nizwa Hospital.

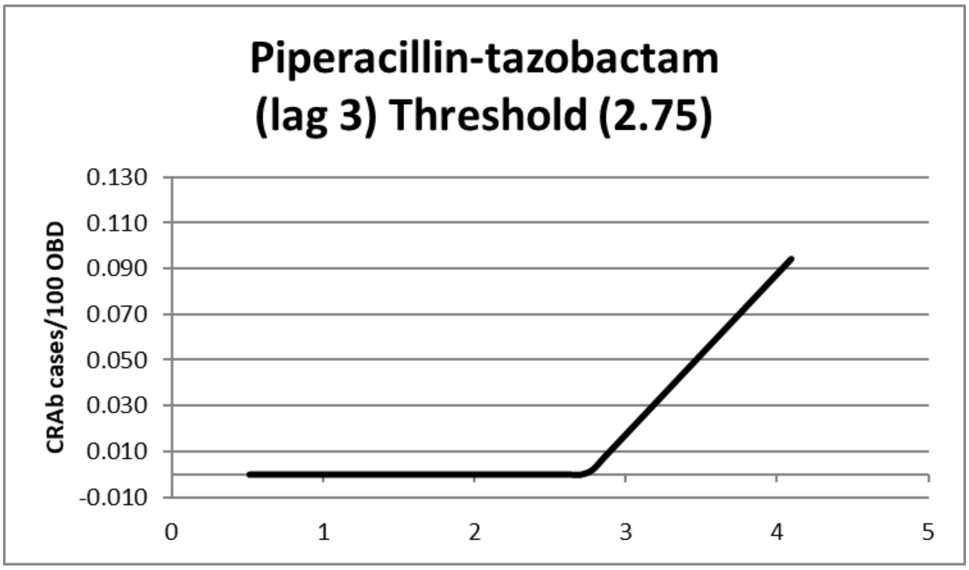

(A)

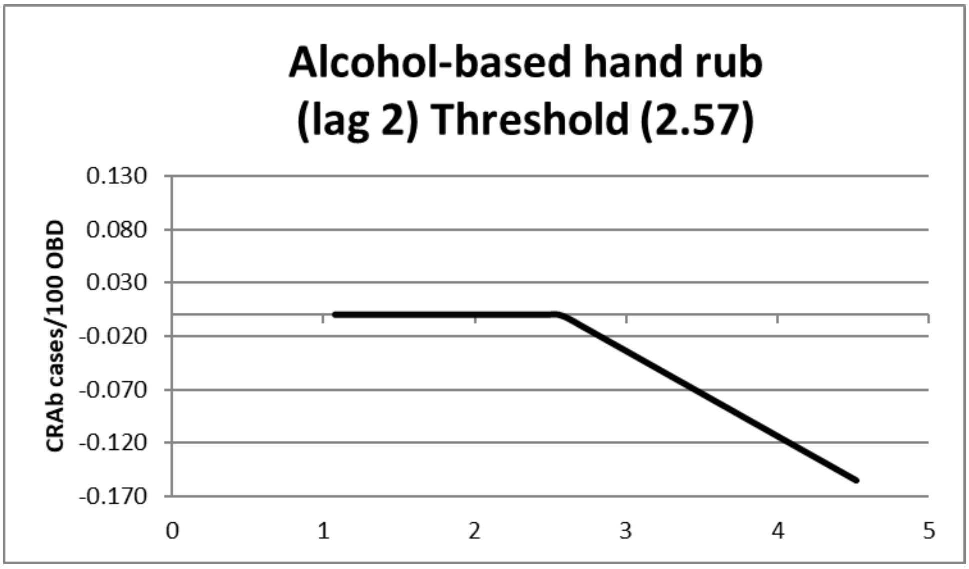

(B)

Figure S5. Contribution charts illustrate the relationship between piperacillin-tazobactam (**A**), alcohol-based hand rub (**B**), and the incidence of CRAb, showing the estimated effect when use levels exceed their respective threshold value, Sur Hospital.

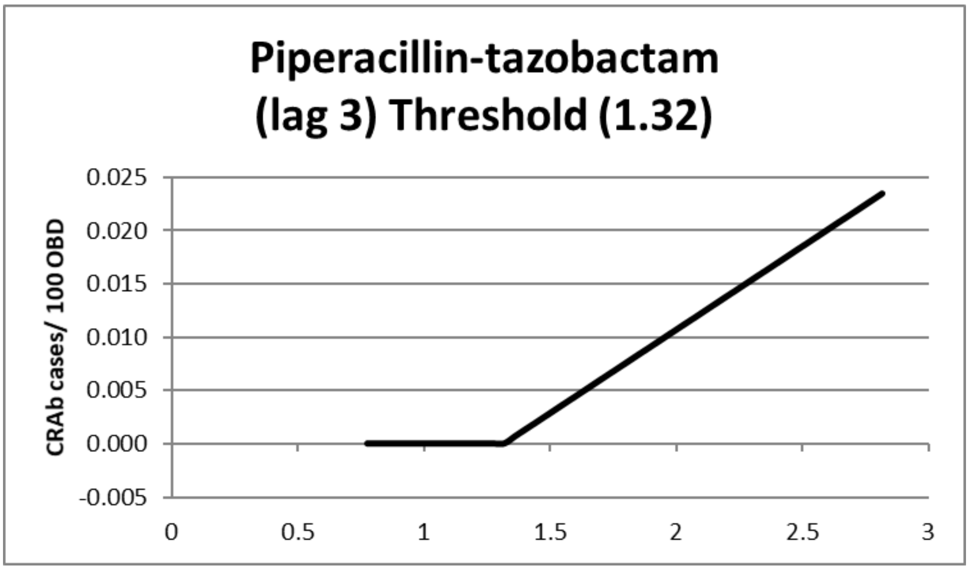

Figure S6. Contribution charts illustrate the relationship between piperacillin-tazobactam and the incidence of CRAb, showing the estimated effect when use levels exceed their respective threshold value, Ibra Hospital.

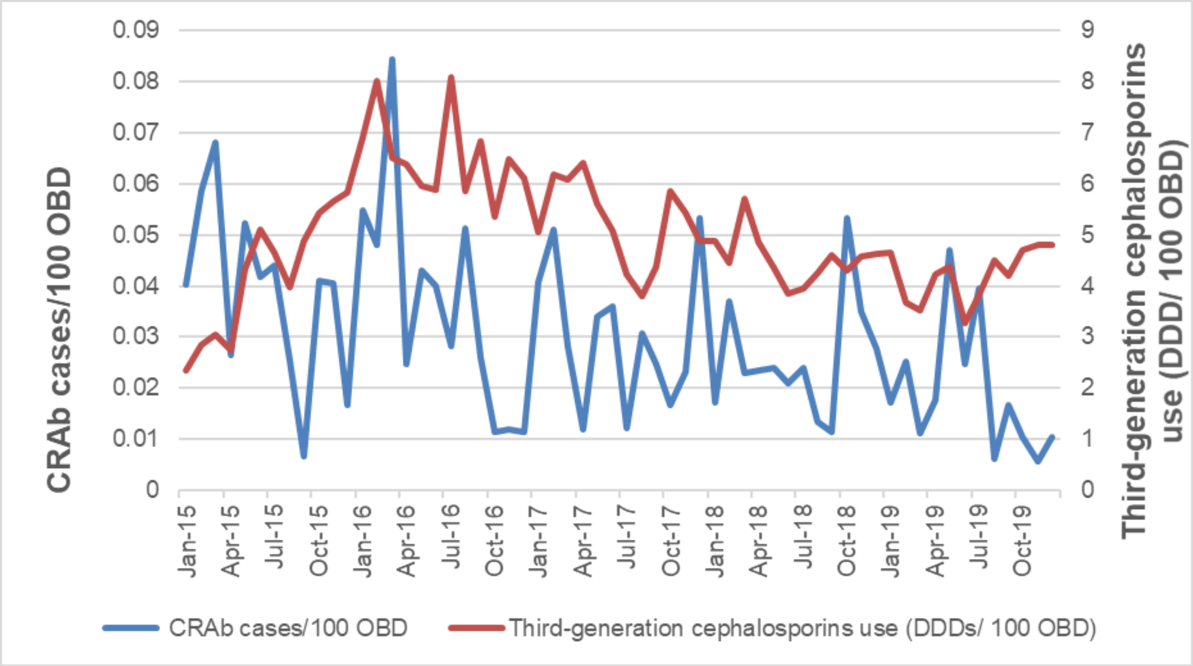

(A)

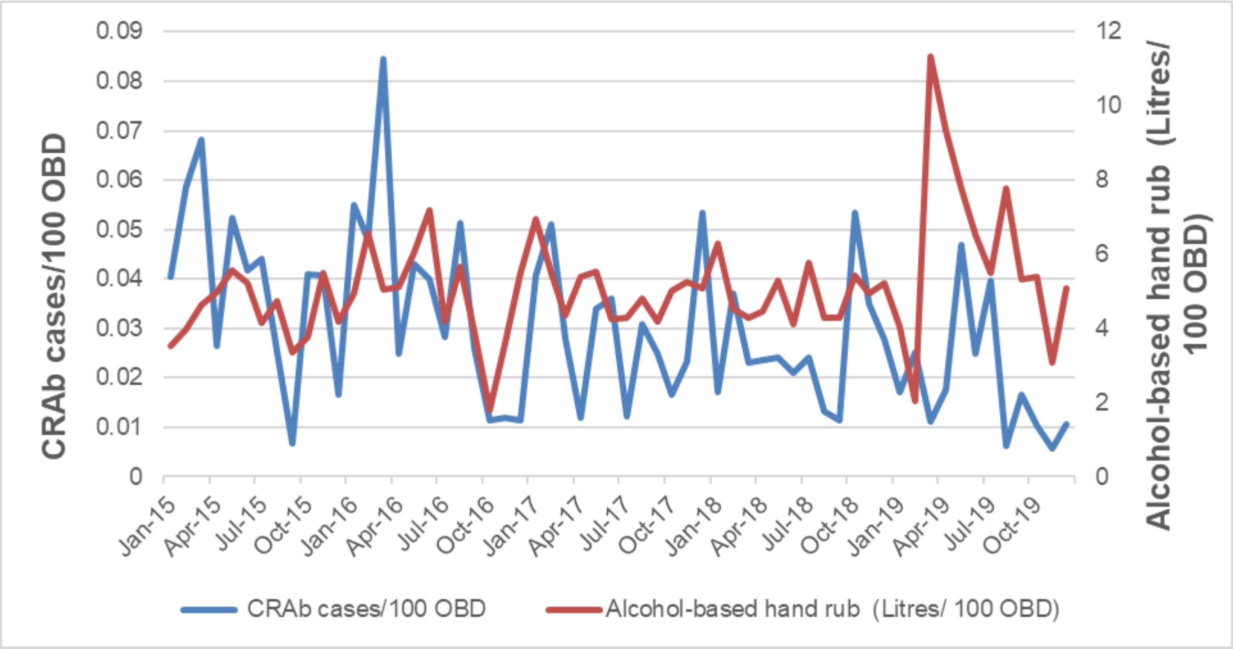

(B)

Figure S7. Monthly incidence of carbapenem-resistant Acinetobacter baumannii (CRAb) versus third-generation cephalosporins use (**A**), and use of alcohol-based hand rub (**B**), Royal Hospital, January 2015-December 2019.

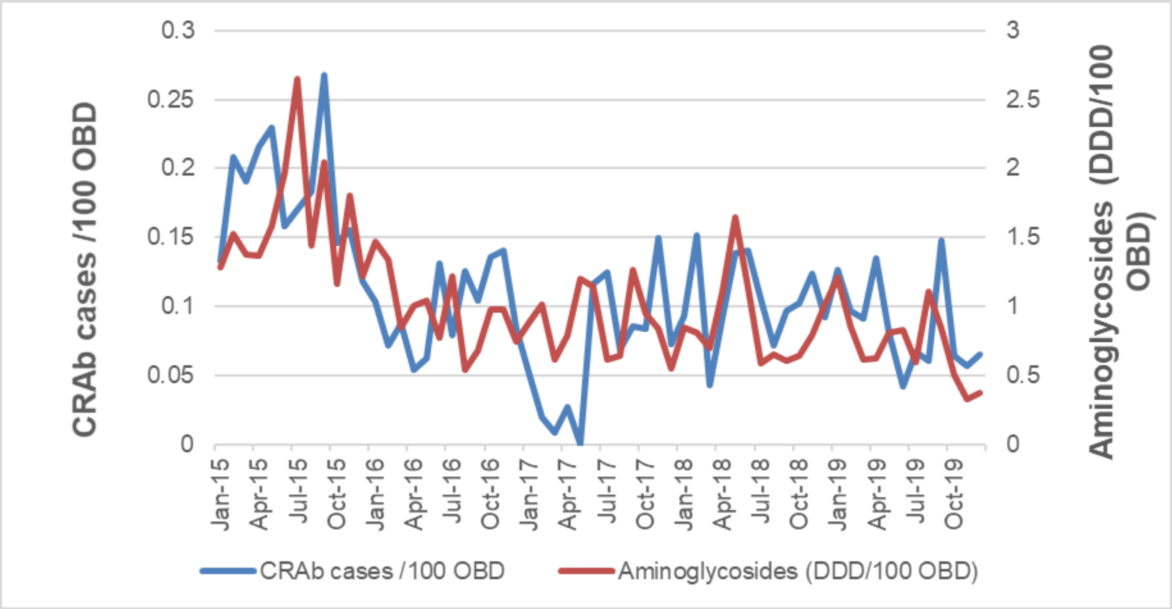

(A)

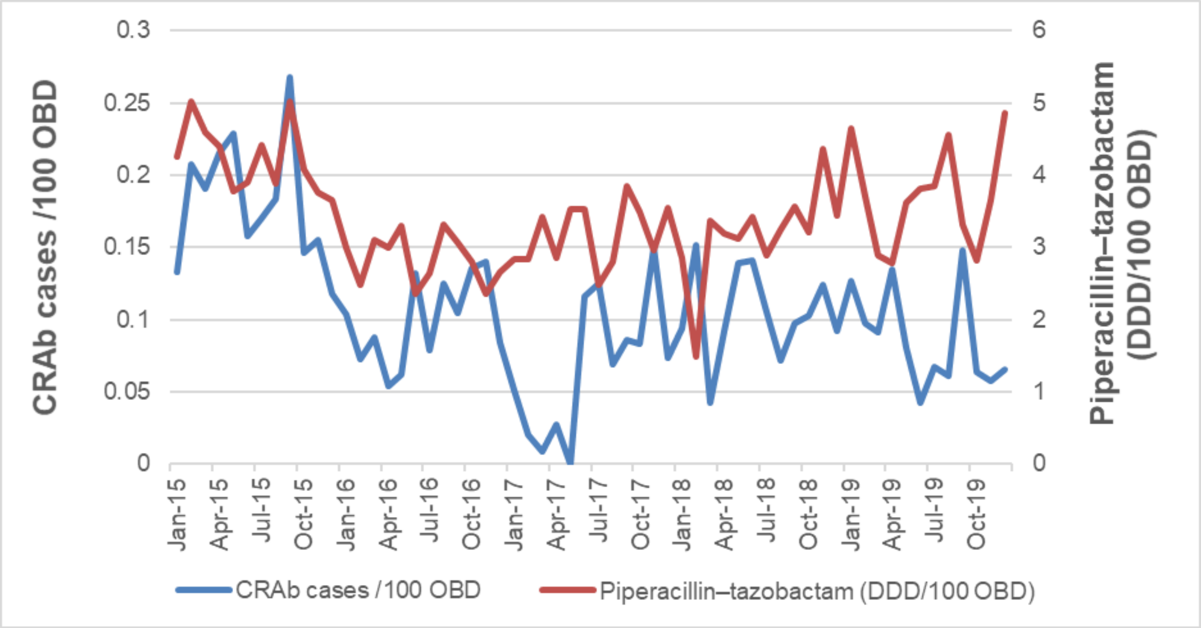

(B)

Figure S8. Monthly incidence of carbapenem-resistant Acinetobacter baumannii (CRAb) versus use of aminoglycosides (**A**), and piperacillin–tazobactam (**B**), Khawlah Hospital, January 2015-December 2019.

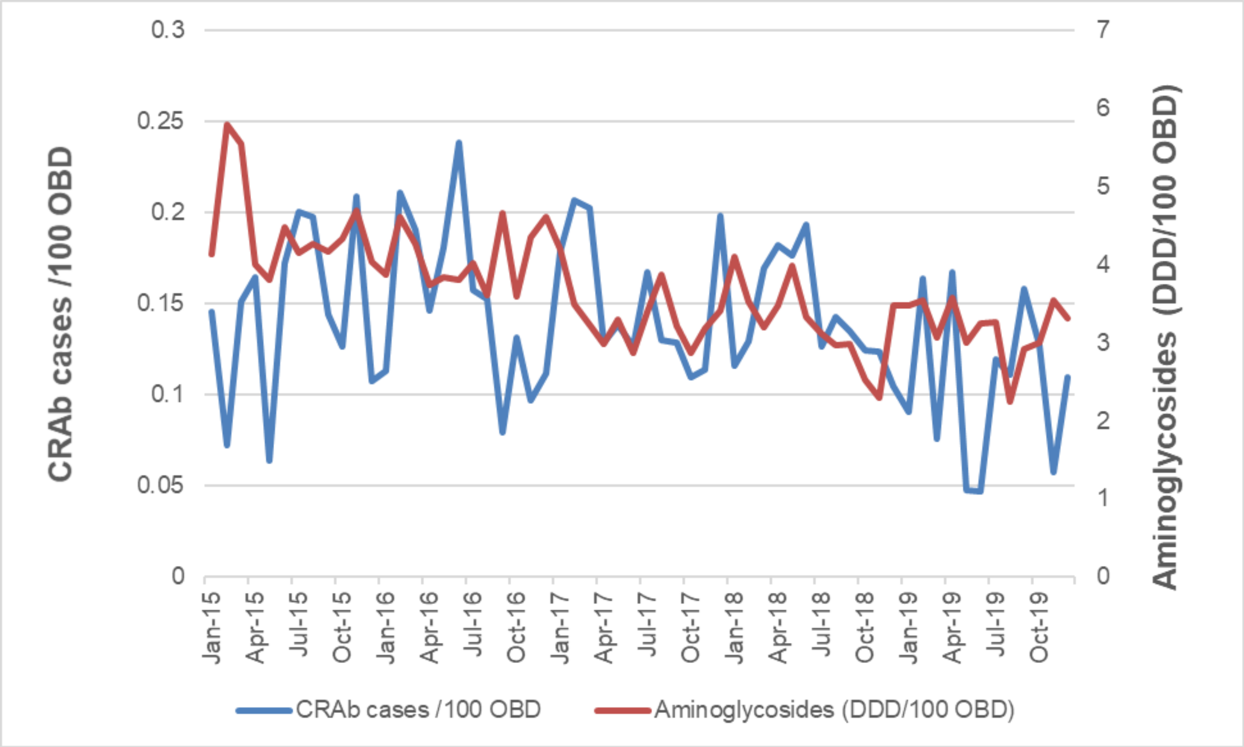

(A)

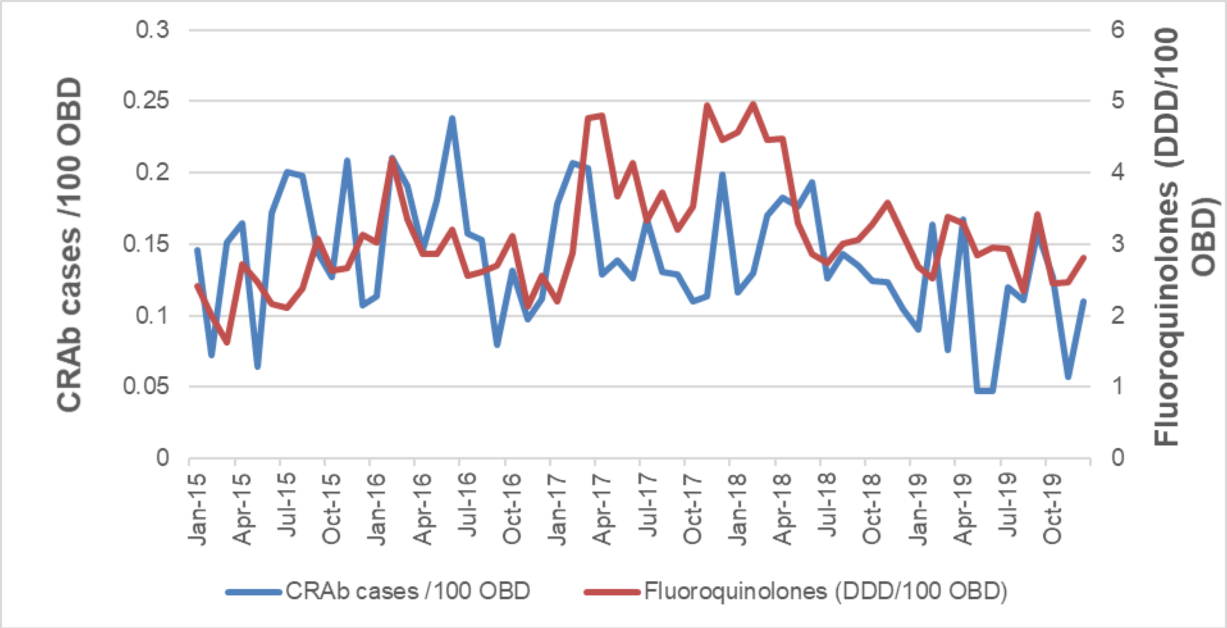

(B)

Figure S9. Monthly incidence of carbapenem-resistant Acinetobacter baumannii (CRAb) versus use of aminoglycosides (**A**), and fluoroquinolones (**B**), As Sultan Qaboos Hospital, January 2015-December 2019.

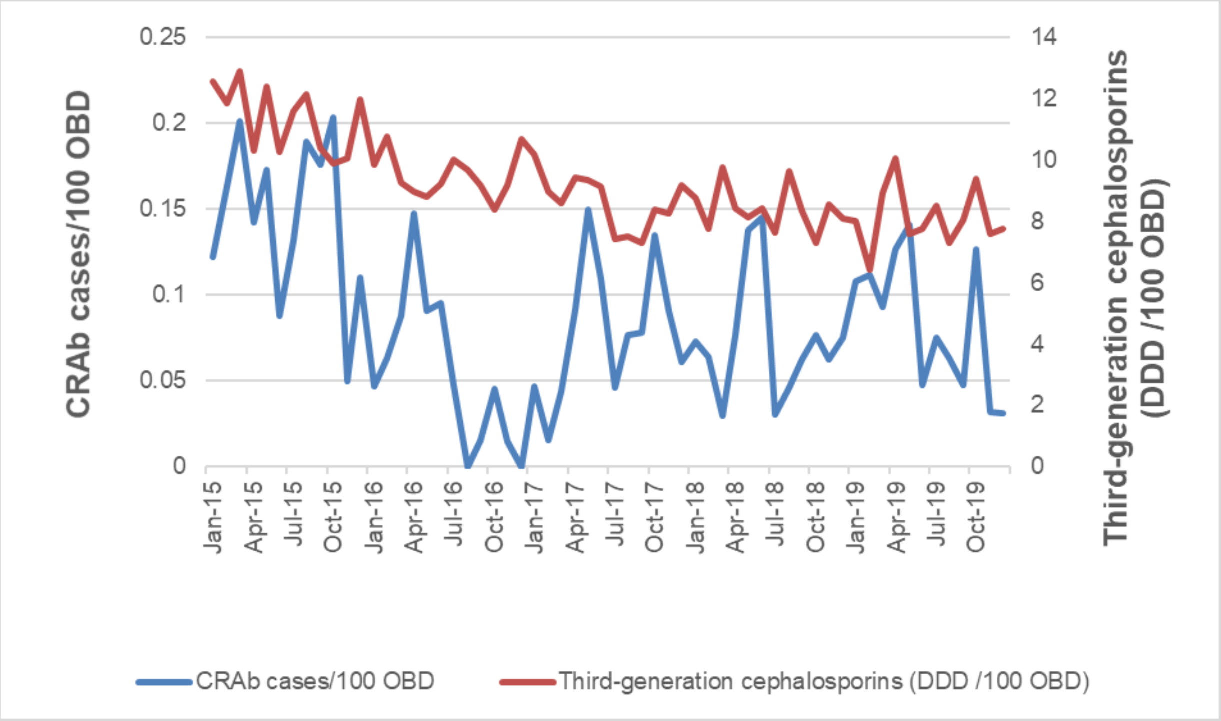

(A)

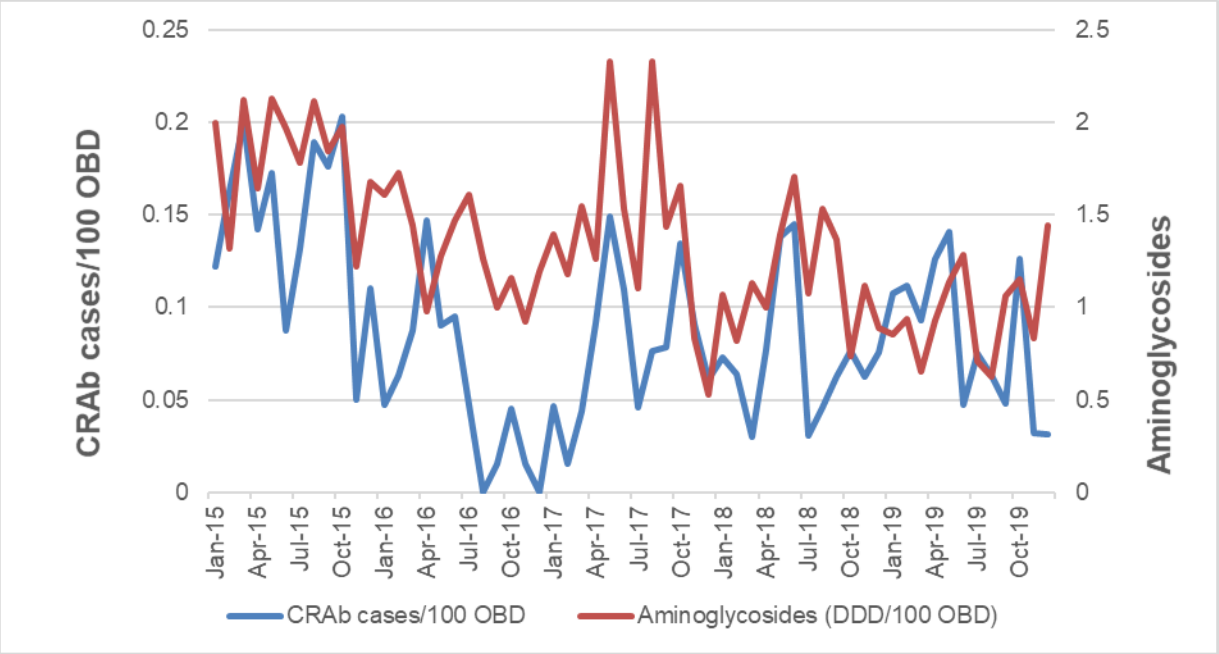

(B)

Figure S10. Monthly incidence of carbapenem-resistant Acinetobacter baumannii (CRAb) versus third-generation cephalosporins (**A**), and aminoglycosides use (**B**), Nizwa Hospital, January 2015-December 2019.

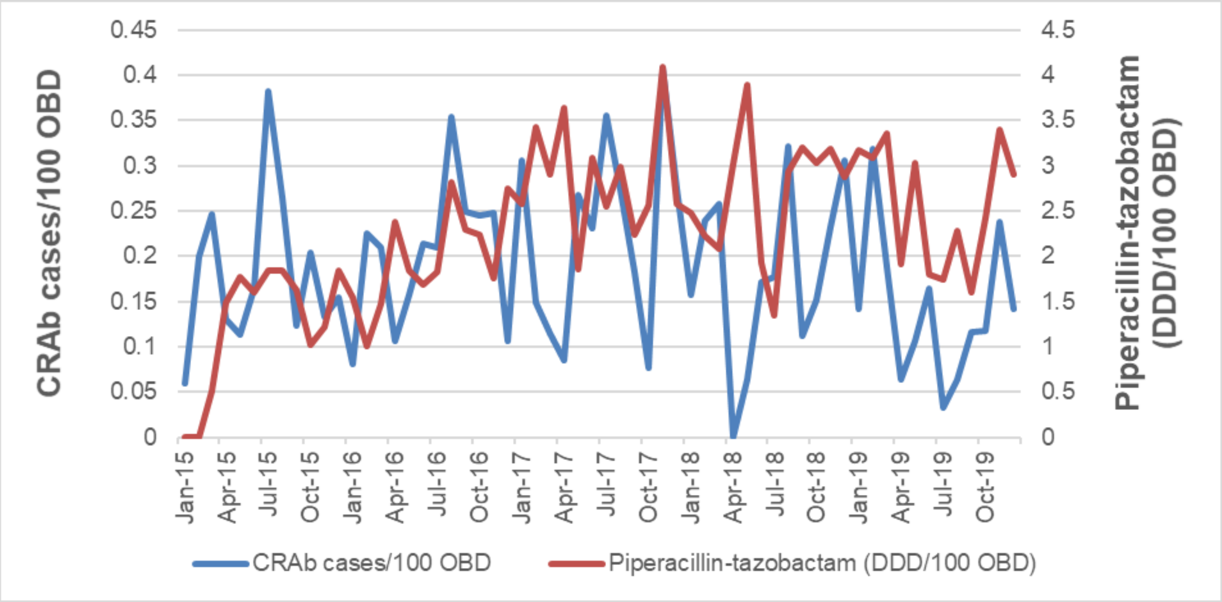

(A)

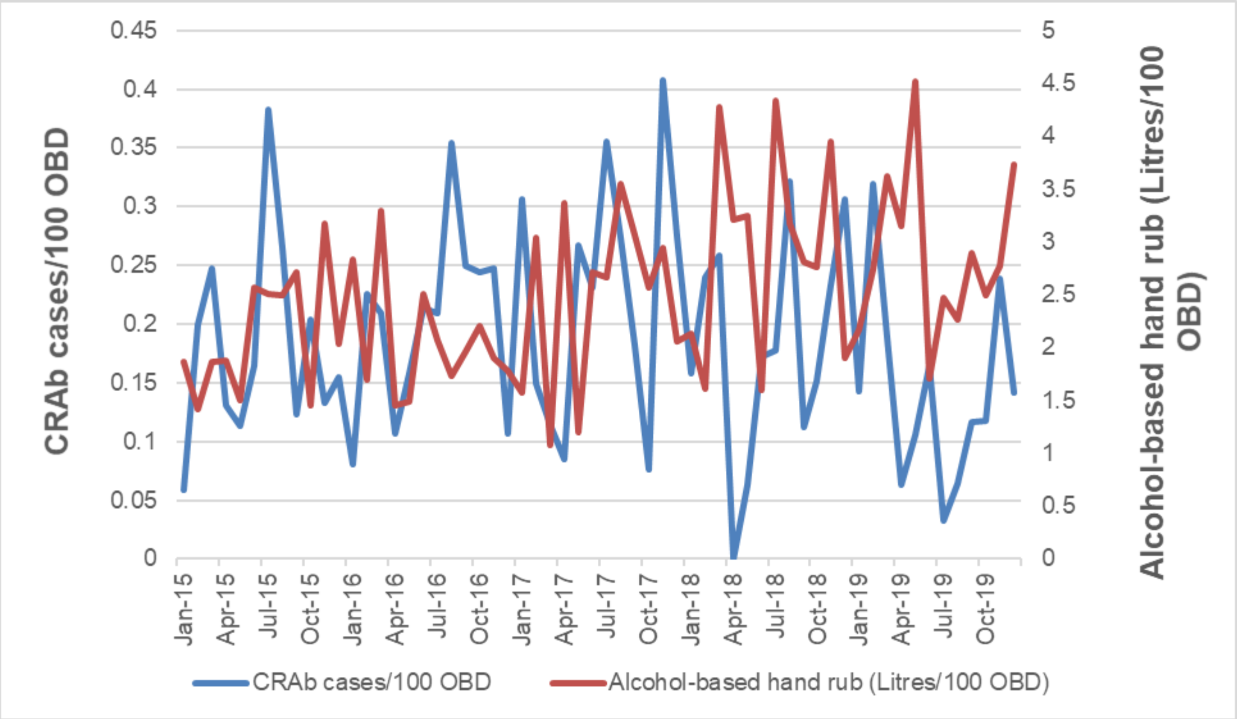

(B)

Figure S11. Monthly incidence of carbapenem-resistant Acinetobacter baumannii (CRAb) versus piperacillin-tazobactam use (**A**), and alcohol-based hand rub use (**B**), Sur Hospital, March 2015-December 2019.

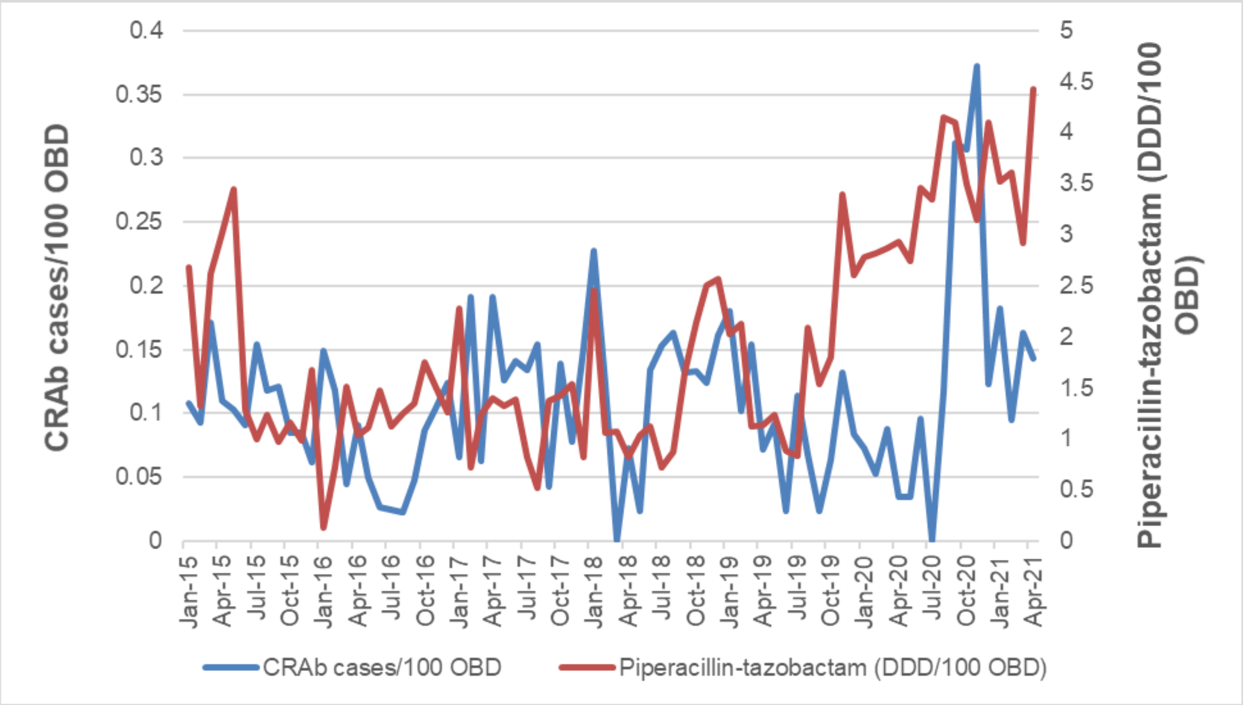

Figure S12. Monthly incidence of carbapenem-resistant Acinetobacter baumannii (CRAb) versus piperacillin-tazobactam use, Ibra Hospital, January 2015-December 2019.

Table S3. General Characteristics of antibiotic stewardship and infection control in participating hospitals from Oman

	Antibiotic stewardship (AMS)	National antimicrobial Guideline was launched in May 2016.	Infections control measurement Following the Ministry of health infection control department policy
Royal Hospital	Yes<br>ABX restriction:<br>Meropenem, colistin, vancomycin, linezolid, tigecycline, clindamycin, ciprofloxacin, liposomal amphotericin, echinocandin, voriconazole<br><br>AMS rounds	Royal Hospital clinical guidelines for CAP/Meningitis/ SA bacteremia/ UTI / CDI/ Sepsis<br>National guidelines are not used frequently	Hand hygiene activities including audits and an orientation program in 2016<br>Isolation policy in 2009<br>MDRO surveillance program in 2013<br>Opening cohort ward for MDRO in 1/2017
Khawlah Hospital	Yes; clinical pharmacist effort was done to check the antibiotic prescriptions and consult with the microbiologist. Restriction of some antibiotics (colistin, meropenem) unless discussed with the teams. PPS was done and share the result with each department. Antibiotic stewardship round.	Yes; some of the departments do not frequently follow the national guidelines and use the international guidelines	Hand hygiene rounds regularly.<br>MDRO surveillance program<br>Isolation policy<br>Infection control ward rounds
As Sultan Qaboos Hospital	No	No, not all departments follow the national guidelines	Hand hygiene audit<br>Isolation of positive cases<br>Active surveillance
Nizwa hospital	No formal committee but both clinical pharmacists and a	Yes; antibiotic national clinical guidelines are followed in the	Active surveillance<br>Hand hygiene

	clinical microbiologist work as advisors to prescribers regarding the choice and dosages of antibiotics.	hospital. Also, the international guidelines serve as a guide whenever national guidelines do not provide sufficient recommendations.	Isolation of positive cases.<br>Infection control ward rounds<br>Emphasis on the use of standard precautions for all cases.<br>Ensuring the following of proper use of personal protective measures for isolated cases.
Sur Hospital	Yes, It is announced in August 2019, before that it was active by the efforts of ID Physician and Clinical Pharmacist. No major intervention was done.	No, only one department is following the guidelines.	Active surveillance<br>Hand hygiene<br>Isolation of positive cases.<br>Infection control ward rounds
Ibra Hospital	Yes, started in December 2020<br>The intervention done by the committee was to shift the use of ciprofloxacin and cefuroxime to nitrofurantoin in UTI patients.	Not all departments follow.	Hand hygiene audit regularly<br>Isolation of positive cases.<br>Active surveillance

CAP: Community-acquired pneumonia, SA: Staphylococcus aureus, UTI: Urinary tract infection, CDI: Clostridioides difficile infection.
